# RNA sequencing reveals the expression profiles of circRNA and indicates that circDDX17 acts as a tumor suppressor in colorectal cancer

**DOI:** 10.1186/s13046-018-1006-x

**Published:** 2018-12-27

**Authors:** Xiang-Nan Li, Zhen-Jun Wang, Chun-Xiang Ye, Bao-Cheng Zhao, Zhu-Lin Li, Yong Yang

**Affiliations:** Department of General Surgery, Beijing Chao-Yang Hospital, Capital Medical University, No. 8 Gongtinan Road , Chaoyang District, Beijing, 100020 People’s Republic of China

**Keywords:** Colorectal cancer, Circular RNAs, High-throughput sequencing, Bioinformatic analysis, CircDDX17, Tumor suppressor

## Abstract

**Background:**

Circular RNA (circRNA) is a novel class of noncoding RNAs with functions in various pathophysiological activities. However, the expression profiles and functions of circRNAs in colorectal cancer (CRC) remain largely unknown.

**Methods:**

High-throughput RNA sequencing (RNA-seq) was performed to assess circRNA expression profiles in 4 paired CRC tissues, and significantly dysregulated circRNAs were validated by quantitative real-time polymerase chain reaction (qRT-PCR). Gene Ontology (GO) and Kyoto Encyclopedia of Genes and Genomes (KEGG) pathway enrichment analyses were performed to predict the potential functions of dysregulated circRNAs. Target miRNAs of circRNAs were predicted using miRanda software, and were further analyzed combining DIANA-miRPath v.3 platform (Reverse Search module) with KEGG pathways of COLORECTAL CANCER and MicroRNAs in cancer (Entry: map05210 and map05206). CircRNA-miRNA interaction networks were constructed using Cytoscape software. Expression levels of a significantly down-regulated circRNA, circDDX17 (hsa_circ_0002211), was detected by qRT-PCR in 60 paired CRC tissues. CircDDX17 was knockdown by siRNA, and the biological functions of circDDX17 were examined in CRC cell lines.

**Results:**

Totally 448 differentially expressed circRNAs were identified, including 394 up-regulated and 54 down-regulated circRNAs. qRT-PCR validation confirmed the reliability of the RNA-Seq data. GO and KEGG analyses revealed that these dysregulated circRNAs were potentially implicated in CRC pathogenesis. Analyses by combining miRanda and miRPath softwares with KEGG pathways suggested that the miRNAs targeted by the top 10 dysregulated circRNAs were associated with the KEGG pathways of COLORECTAL CANCER and MicroRNAs in cancer, indicating that circRNA-miRNA interactions might play important functional roles in the initiation and progression of CRC. The results of qRT-PCR for circDDX17 in 60 paired CRC tissues showed that circDDX17 was significantly down-regulated in CRC tissues and associated with unfavorable clinicopathological parameters. In vitro experiments showed that silencing of circDDX17 promoted CRC cell proliferation, migration, invasion, and inhibited apoptosis.

**Conclusions:**

In conclusion, we have identified numerous circRNAs that are dysregulated in CRC tissues compared with adjacent normal mucosa tissues. Bioinformatic analyses suggested that these dysregulated circRNAs might play important functional roles in CRC tumorigenesis. CircDDX17 functions as a tumor suppressor and could serve as a potential biomarker and a therapeutic target for CRC.

**Electronic supplementary material:**

The online version of this article (10.1186/s13046-018-1006-x) contains supplementary material, which is available to authorized users.

## Background

According to the latest data in 2018, colorectal cancer (CRC) ranked the second cause of cancer death (9.2% of the total cancer deaths), and was the third most commonly diagnosed cancer (10.2% of the total cases) [1]. Despite considerable progresses have been made in the diagnosis and therapy of this disease, the mortality of CRC is still high, which warrants the necessity to explore other unknown mechanisms contributing to CRC tumorigenesis.

Circular RNAs (circRNAs) represent a novel class of endogenous noncoding RNAs which do not have 5′ or 3′ ends but are covalently linked to form a closed circular structure [2]. CircRNAs are widely expressed in diverse cell types and species; more than 20,000 circRNAs have been found in eukaryotes [3]. CircRNAs differ from other RNAs because they are characterized by their covalently closed loop structures without 5′ caps and 3′ poly(A) tails [4]. This closed loop structure, which is also called a “back-splicing” structure, is generated from the joining of an upstream 3′ splice acceptor to a downstream 5′ splice donor [5]. Due to the special circular covalently bonded structure, circRNAs are resistant to exonucleases, which makes circRNAs more stable than linear RNA isoforms [2]. Circular RNA arises from exonic, intronic and intergenic regions [6]. The exonic circRNAs (only having exon sequences) are the most abundant circRNAs and mainly reside in the cytoplasm, whereas intron-retaining circRNAs generally reside in nuclei [7]. Up to now, the exonic circRNAs are the most studied types of circRNAs.

Many of the functions of circRNAs have been elucidated over the past few years. For example, circRNAs can regulate transcription and alternative splicing, interact with RNA binding proteins (RBPs), and function as microRNA (miRNA) sponges [8, 9]. Based on the functions of circRNAs, many studies have investigated the roles of circRNAs in physiology and pathology, and have confirmed that circRNAs play important roles in the initiation and progression of human diseases, especially cancers [10–13]. These observations indicate that circRNAs may be a new kind of potential biomarkers and therapeutic targets for cancer. However, as most circRNAs remain largely unexplored, further research is needed to elucidate their functions.

In this study, by using high-throughput RNA sequencing (RNA-seq), we explored the expression profiles of circRNAs and identified 448 significantly dysregulated circRNAs in CRC compared with normal mucosa tissues. Expression levels of 10 circRNAs selected from the top 10 up- and down-regulated (top 10 dysregulated) circRNAs were measured by quantitative real-time polymerase chain reaction (qRT-PCR) in CRC tissues, and the results confirmed the RNA-seq data. Gene Ontology (GO) and Kyoto Encyclopedia of Genes and Genomes (KEGG) pathway enrichment analyses revealed the potential roles of circRNAs in CRC. Analyses by combining miRanda and miRPath softwares with KEGG pathways suggested that the miRNAs targeted by the top 10 dysregulated circRNAs were associated with the KEGG pathways of COLORECTAL CANCER and MicroRNAs in cancer, indicating that circRNA-miRNA interactions might play important functional roles in the initiation and progression of CRC. We further tested circDDX17 (hsa_circ_0002211) in 60 pairs of CRC samples by qRT-PCR and the results showed that circDDX17 was decreased in CRC tissues and associated with unfavorable clinicopathological parameters. In vitro experiments showed that silencing of circDDX17 could promote CRC cell proliferation, migration, invasion, and inhibit apoptosis. We have demonstrated that circRNAs are dysregulated in CRC and play important roles in the progression of CRC.

## Methods

### Patients and specimens

A total of 60 pairs of CRC and adjacent normal mucosa tissues were collected from patients undergone surgery between September 2016 and February 2018 at the Department of General Surgery, Beijing Chao-Yang Hospital, Capital Medical University (Beijing, China). The specimens were immediately frozen in liquid nitrogen after collection and stored at − 80 °C until RNA extraction. Among the 60 pairs of CRC tissues, 4 pairs were selected for RNA-seq, 20 pairs (not including the tissues for RNA-seq) were used for qRT-PCR to validate the RNA-seq data, and all the 60 pairs were used for qRT-PCR to detect the level of circDDX17. All of the specimens were verified by histopathology. None of these patients received preoperative chemotherapy, radiotherapy, or targeted therapy.

### Cell lines

The human CRC cell lines SW480, SW620, HT29, LoVo, HCT116, and RKO were purchased from the American Type Culture Collection (Manassas, VA, USA). Cells were cultured in Dulbecco’s Modified Essential Medium (Invitrogen, Carlsbad, CA, USA) supplemented with 10% fetal bovine serum (FBS; Gibco, NY, USA), 100 U/ml penicillin and 100 μg/ml streptomycin (Gibco, NY, USA) at 37 °C in 5% humidified CO_2_ atmosphere.

### RNA sequencing

Total RNAs were extracted with Trizol (Invitrogen, Carlsbad, CA, USA). RNA integrity was analyzed with Agilent 2100 Bioanalyzer (Agilent Technologies, Santa Clara, CA, USA). RNA concentration was tested by Qubit RNA Assay Kit in Qubit Fluorometer (Invitrogen, Carlsbad, CA, USA). Total RNA samples that meet the following requirements were used in subsequent experiments: RNA integrity number (RIN) ≥ 7.0 and a 28S:18S ratio ≥ 1.5.

Sequencing libraries were generated and sequenced by CapitalBio Technology (Beijing, China). A total amount of 5 μg RNA per sample was used. Briefly, total RNAs were subjected to ribosomal RNA (rRNA) removal using the Ribo-Zero™ Magnetic Kit (Epicentre Technologies, Madison, WI, USA). To remove linear RNAs, total RNAs were digested with RNase R (Epicentre Technologies, Madison, WI, USA). The NEBNext Ultra RNA Library Prep Kit for Illumina (NEB, USA) was used to construct the libraries for sequencing according to the manufacturer’s protocol. The RNA was then fragmented into pieces of ~ 300 base pairs (bp) in length in NEBNext First Strand Synthesis Reaction Buffer (5x). The first-strand cDNA was synthesized from the RNA fragments by reverse transcriptase and random hexamer primers, and the second-strand cDNA was synthesized in Second Strand Synthesis Reaction Buffer with dUTP Mix (10x). The end of the cDNA fragment was subjected to an end repair process which included the addition of a single “A” base, followed by ligation of the adapters. After the ligation of Illumina sequencing adaptors, the second strand of cDNA was digested using the USER Enzyme (NEB, USA) to construct a chain specific library. To amplify the library DNA, libraries were purified and enriched by PCR. Then, the libraries were qualified by Agilent 2100 and quantified using KAPA Library Quantification kit (KAPA Biosystems, South Africa). Finally, the libraries were subjected to paired-end sequencing with pair end 150 bp reading length on an Illumina HiSeq sequencer (Illumina, San Diego, CA, USA).

### Bioinformatic analyses

FastQC software (v0.11.2) [14] was used to assess the sequencing quality of raw data of fastq format. Low quality data were filtered using NGSQC software (v2.3.2) [15]. The clean reads with high quality were then aligned to the human reference genome (GRCh38/hg38) using Tophat2 software (v2.0.13) [16] with default parameters. The sequencing data that cannot aligned to reference genome directly were subjected to the subsequent circRNA analysis by recognition of the reverse splicing event using Find_circ (v1.0) [17] and CIRCexplorer2 softwares [18]. Limma (v3.32.10) [19] package of R software was used to analyze the different expression of circRNAs. Function annotation and pathway enrichment analyses for the host genes of differentially expressed circRNAs were performed with KOBAS 3.0 [20].

### Analyses of circRNA-miRNA interaction in CRC

Target miRNAs of circRNAs were predicted using miRanda software (v3.3a) [21]. Detailed setting parameters of miRanda software were as follows: Gap Open Penalty: − 9; Gap Extend Penalty: − 4; Score Threshold: 140; Energy Threshold: − 20 kcal/mol; Scaling Parameter: 4. The miRNAs associated with the KEGG pathway of COLORECTAL CANCER (Entry: map05210; https://www.kegg.jp/) were analyzed using DIANA-miRPath v.3 platform (Reverse Search module) [22]. Furthermore, we also screened target miRNAs of top 10 dysregulated circRNAs according to the KEGG pathway of MicroRNAs in cancer (section of “Colorectal cancer”; Entry: map05206; https://www.kegg.jp/), and target miRNAs associated with MicroRNAs in cancer pathway were identified. The maps of circRNA-miRNA interaction network were constructed using Cytoscape software [23].

### Quantitative real-time PCR

Total RNAs from cells and tissue samples were isolated using Trizol (Invitrogen, Carlsbad, CA, USA). Specific divergent primers spanning the back-splice junction site of circRNAs were designed. To quantify the amount of mRNA and circRNA, cDNA was synthesized using PrimeScript™ RT reagent Kit (TaKaRa, Dalian, China). The real-time PCR analyses were performed using TB Green™ *Premix Ex Taq*™ II (TaKaRa, Dalian, China) and an ABI 7500 real-time PCR system (Applied Biosystems, Foster City, CA, USA). 18 s rRNA was employed as internal control. The relative expression of RNAs was calculated by 2^−ΔΔCt^ method. All the primers (listed in Additional file 1: Table S1) were synthesized by Sangon Biotech (Shanghai, China).

### Transfection

Small interfering RNAs (siRNAs) targeting the back-splice junction sites of circDDX17 and negative control oligonucleotide (Additional file 1: Table S2) were designed and synthesized by GenePharma (Shanghai, China). Transfections were performed with final concentrations of 50 nM of siRNAs using the Lipofectamine 3000 reagent (Invitrogen, Carlsbad, CA, USA) according to the manufacturer’s instructions.

### Colony formation assay

For colony formation assay, 24 hours after transfection, SW480 and SW620 cells were seeded into 6-well plates and then incubated for about two weeks. Then the colonies were fixed with 4% paraformaldehyde for 15 min and stained with 0.1% crystal violet for 15 min at room temperature. The cell colonies were counted and photographed.

### CCK-8 assay

Cell proliferation assay was performed using CCK-8 kit (Dojindo Laboratories, Kumamoto, Japan) following the manufacturer’s instructions. Approximately 1 × 10^3^ transfected SW480 and SW620 cells in 100 μl were incubated in 96-well plates. At 0, 24, 48, 72 and 96 h, cells in each well were treated with the CCK-8 reagent (10 μl) and incubated at 37 °C for 2 h. The optical density (OD) at 450 nm was read using a Varioskan Flash Spectral Scanning Multimode Reader (Thermo Fisher Scientific, Waltham, MA, USA). Cells in each group were tested for 5 replicates.

### Apoptosis analysis

Cell apoptosis was analyzed using the Annexin V-FITC Apoptosis Detection Kit (KeyGen Biotech, Nanjing, China) according to the manufacturer’s instructions. 48 h after transfection, SW480 and SW620 cells were stained with FITC and propidium iodide. Then flow cytometry was performed using a FACS Canto II (BD Biosciences) and the data were analyzed using BD FCSDiva Software and FCS Express 5 software (De Novo Software, Los Angeles, CA).

### Cell migration and invasion assays

Cell migration and invasion assays were conducted using Transwell Permeable Supports, Polycarbonate (PC) Membrane (Corning, NY, USA), which was coated with (for invasion assays) or without (for migration assays) the matrigel (Corning, NY, USA) according to the manufacturer’s instructions. 24 h after transfection, cells in 200 μl serum-free medium were seeded into the upper compartment, with 700 μl culture medium containing 20% FBS in the lower compartment. After 24 h incubation, the cells located on the upper surfaces of the transwell chamber were erased with cotton swabs and the cells located on the lower surfaces were fixed with 4% paraformaldehyde for 15 min and stained with 0.1% crystal violet for 15 min at room temperature. The stained cells were photographed and counted in five randomly selected fields under a Leica DM4000B microscope (Leica, Wetzlar, Germany).

### Statistical analysis

Statistical analyses were performed using SPSS 23.0 (IBM, SPSS, Chicago, IL, USA) and figures were produced using GraphPad Prism 7.0 (GraphPad Software, Inc. La Jolla, CA, USA). Comparisons between groups were analyzed by Student’s t test, Wilcoxon signed rank test, and One-way analysis of variance (one-way ANOVA), as appropriate. The categorical data were analyzed by Chi-square test. Data were presented as the mean ± s.d. of at least three independent experiments. A *p* value < 0.05 was considered statistically significant.

## Results

### Expression profiles of circRNA in CRC

**Fig. 1:**
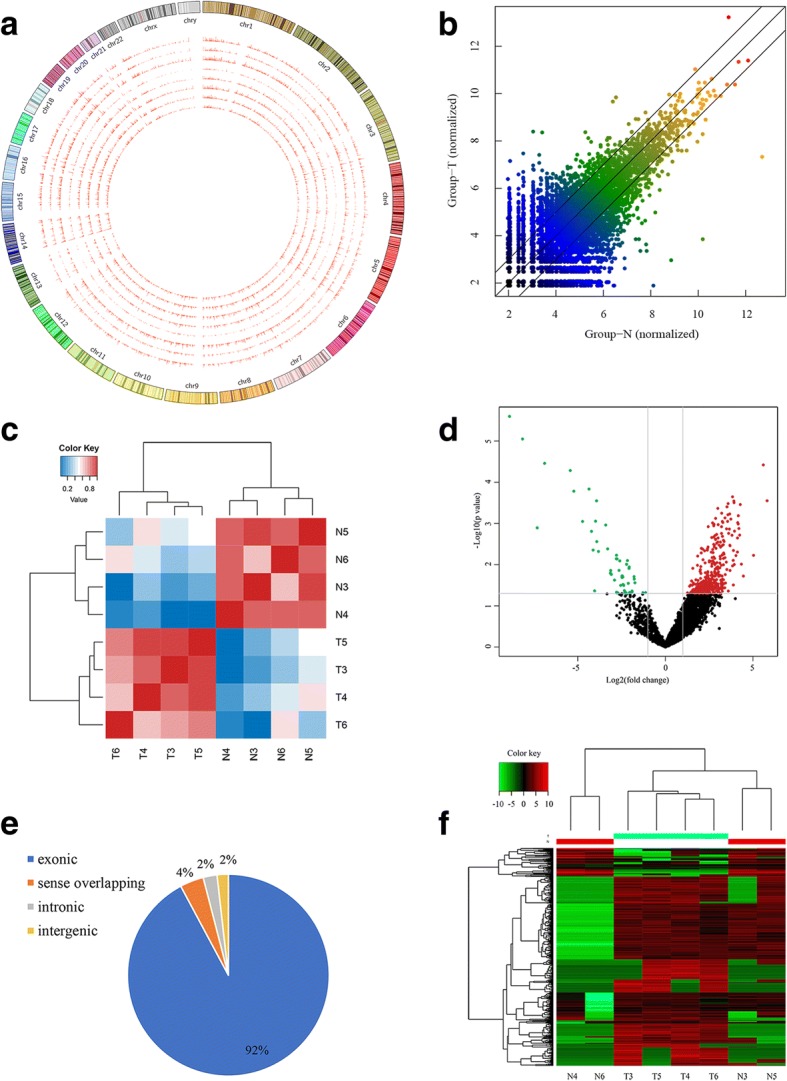
**a** Circos plot showed the locations of cricRNA on human chromosomes. The outermost layer was a chromosome map of the human genome. The inner 8 circles represented all circRNAs of each sample detected by RNA-seq. The inner circles from outside to inside corresponded to sample N3, N4, N5, N6, T3, T4, T5, and T6, respectively. The bar chart presented the expression levels of circRNA. **b** The scatter plot presented the circRNA expression variations between CRC and control groups. The values of the X and Y axes represented the normalized circRNA signal values (log2 scaled). The circRNAs above the top black line and below the bottom black line displayed greater than two-fold change of up- and down-regulation. **c** Heatmap of inter-sample correlation showed there was an obvious difference of significantly dysregulated circRNA expression levels between CRC and control groups, but the difference was slight within each group. The Pearson’s correlation coefficient was represented by a color scale. The intensity increased from blue (relatively lower correlation) to red (relatively higher correlation). Correlation was evaluated by Pearson’s correlation coefficient of significantly dysregulated circRNA expression levels. **d** Volcano plot displayed the dysregulated circRNAs between CRC and control groups. The vertical gray lines corresponded to two-fold up- and down-regulation (log2 scaled), and the horizontal gray line represented a *p* value of 0.05. The red points represented significantly up-regulated circRNAs in CRC, and the green points represented significantly down-regulated circRNAs in CRC. **e** Classification of the significantly dysregulated circRNAs based on genomic origin. **f** Hierarchical clustering of the dysregulated circRNAs in CRC. The expression values were represented by a color scale. The intensity increased from green (relatively lower expression) to red (relatively higher expression). Each column represented one tissue sample, and each row represented a single circRNA. T, CRC tumor tissues; N, adjacent normal mucosa tissues

A total of 21,458 circRNAs were detected by RNA-Seq in 4 pairs of CRC and adjacent normal mucosa tissues. These circRNAs were widely distributed in all chromosomes including sex chromosomes X and Y (Fig. 1a). The scatter plot presented the difference in circRNA expression between the two groups (Fig. 1b). We evaluated Pearson’s correlation coefficient of significantly dysregulated circRNA expression level among different tissues, and the heatmap of inter-sample correlation (Fig. 1c) showed that there was an obvious difference of transcript expression levels between the CRC and control groups, but the difference was slight within each group. After screening of differentially expressed circRNAs by fold-change filtering (|log2(fold change)| > 1) and Student’s t-testing (*p* value < 0.05), 448 differentially expressed circRNAs were identified. Compared with the normal mucosa tissues, there were 394 significantly up-regulated and 54 significantly down-regulated circRNAs in CRC tissues. Volcano plots visualized the significantly differentially expressed circRNAs in CRC tissues (Fig. 1d). Most differentially expressed circRNAs derived from exons (Fig. 1e). 15 circRNAs were identified as new circRNAs that had not been annotated in the circBase or circ2Traits database [24, 25] (Additional files 2 and 3: Datas 1 and 2). The results of hierarchical clustering (Fig. 1f) suggested that the circRNA expression patterns were distinguishable between the CRC and control groups. The top 10 dysregulated circRNAs are listed in Table 1.

**Table 1:** Top 10 dysregulated circRNAs in CRC

CircRNA ID	Log_2_FC^a^	*P* value	regulation	chromosome	circBase ID	Gene Name
circASPHD1	5.80	2.82E-04	up	chr16	hsa_circ_0105346	ASPHD1
circPVT1	5.59	3.81E-05	up	chr8	hsa_circ_0001821	PVT1
circSPIDR	5.03	5.95E-03	up	chr8	hsa_circ_0084188	SPIDR
circFGD6	4.45	1.89E-02	up	chr12	hsa_circ_0099548	FGD6
circHOMER1	4.34	9.73E-03	up	chr5	hsa_circ_0006916	HOMER1
circAPLP2	4.28	3.49E-04	up	chr11	hsa_circ_0000372	APLP2
circCDK8	4.24	1.92E-03	up	chr13	hsa_circ_0029803	CDK8
circMACROD1	4.20	1.33E-03	up	chr11	hsa_circ_0096088	MACROD1
circVAPA	4.16	6.51E-04	up	chr18	hsa_circ_0006990	VAPA
circTRAPPC9	4.16	1.03E-03	up	chr8	hsa_circ_0004380	TRAPPC9
circPRKAG2	−8.90	2.52E-06	down	chr7	–	PRKAG2
circITGAL	−8.15	8.91E-06	down	chr16	–	ITGAL
circITFG2	−7.32	1.28E-03	down	chr12	hsa_circ_0000374	ITFG2
circABTB1	−6.89	3.48E-05	down	chr3	hsa_circ_0067175	ABTB1
circDDX17	−5.43	5.20E-05	down	chr22	hsa_circ_0002211	DDX17
circTRPM4	−5.23	1.65E-04	down	chr19	hsa_circ_0005317	TRPM4
circAMBRA1	−4.73	8.93E-04	down	chr11	hsa_circ_0009155	AMBRA1
circSCAND2P	−4.36	1.46E-04	down	chr15	hsa_circ_0036592	SCAND2P
circLMF1	−4.22	1.55E-03	down	chr16	hsa_circ_0007701	LMF1
circGIGYF1	−4.13	4.42E-03	down	chr7	hsa_circ_0006271	GIGYF1

^a^*FC* fold change

### Validation of circRNA expression

**Fig. 2:**
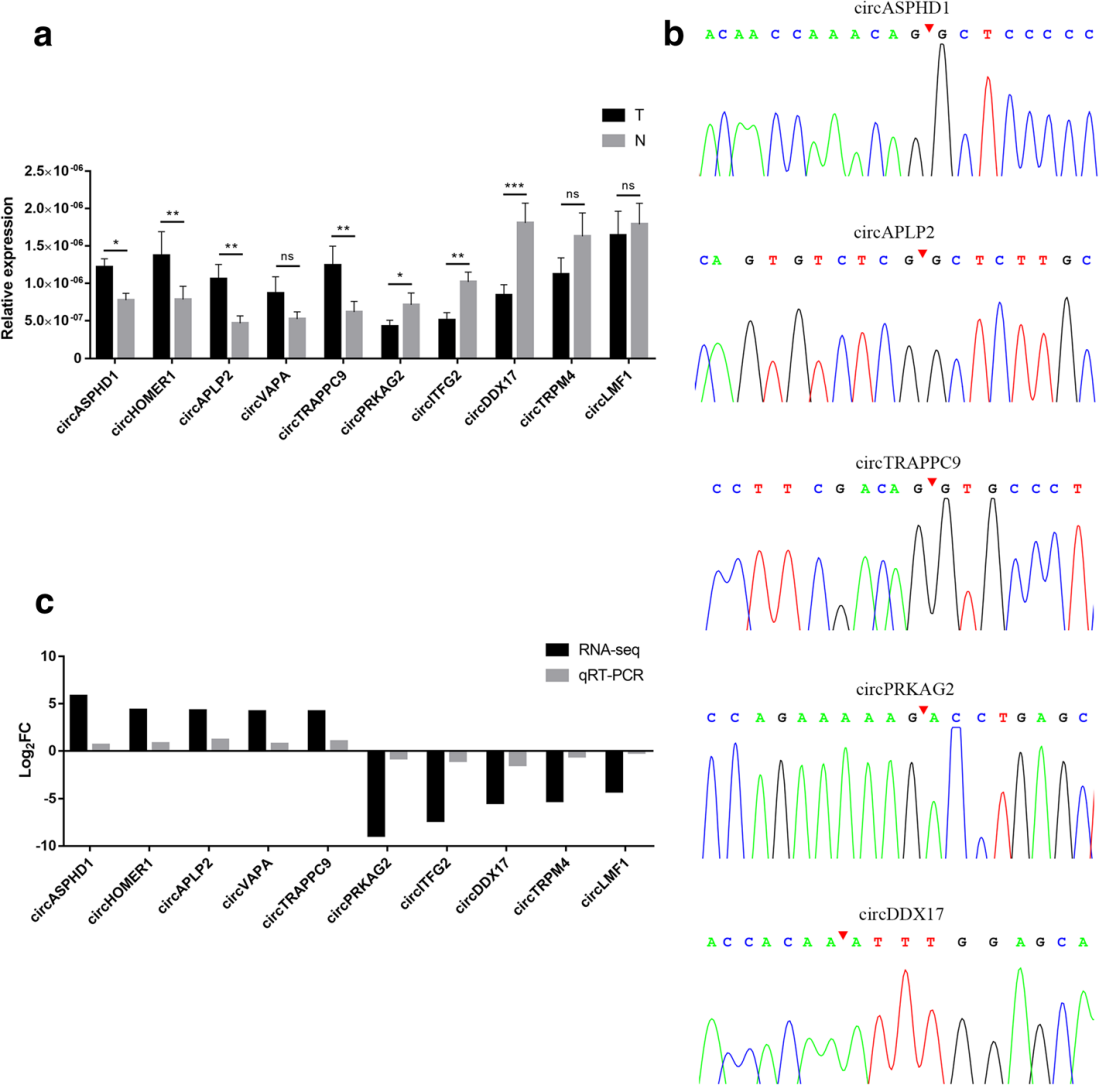
qRT-PCR validation of ten differentially expressed circRNAs in 20 pairs of CRC samples. **a** The relative expression levels of 5 up-regulated and 5 down-regulated circRNAs (selected from the top 10 dysregulated circRNAs) in 20 pairs of CRC and adjacent normal mucosa tissues. **b** Sanger sequencing confirmed the back-splice junction sites of circRNAs. **c.** Comparison of log_2_FC in ten differentially expressed circRNAs between RNA-Seq and qRT-PCR. Data are shown as means ± s.d. of at least three independent experiments. *p < 0.05, **p < 0.01, ***p < 0.001. NS, not significant. T, CRC tumor tissues; N, adjacent normal mucosa tissues

To verify the RNA-Seq data, qRT-PCR was performed for 5 up-regulated and 5 down-regulated circRNAs selected from the top 10 dysregulated circRNAs (two were the most dysregulated, and eight were randomly chosen). The expression levels of those 10 circRNAs were measured in another 20 pairs of CRC tissues. We designed specific divergent primers spanning the back-splice junction sites of circRNAs. The results showed that the expression of circASPHD1, circHOMER1, circAPLP2, circVAPA, and circTRAPPC9 was up-regulated, and the expression of circPRKAG2, circITFG2, circDDX17, circTRPM4, and circLMF1 was down-regulated (Fig. 2a). To verify the back-splice junction sequences, five circRNA qRT-PCR amplified products were sent to Sanger sequencing, and the sequencing results confirmed the back-splice junction sites (Fig. 2b). The qRT-PCR results were highly consistent with the RNA-seq data (Fig. 2c), confirming the reliability of the RNA-Seq results.

### GO and KEGG analyses

**Fig. 3:**
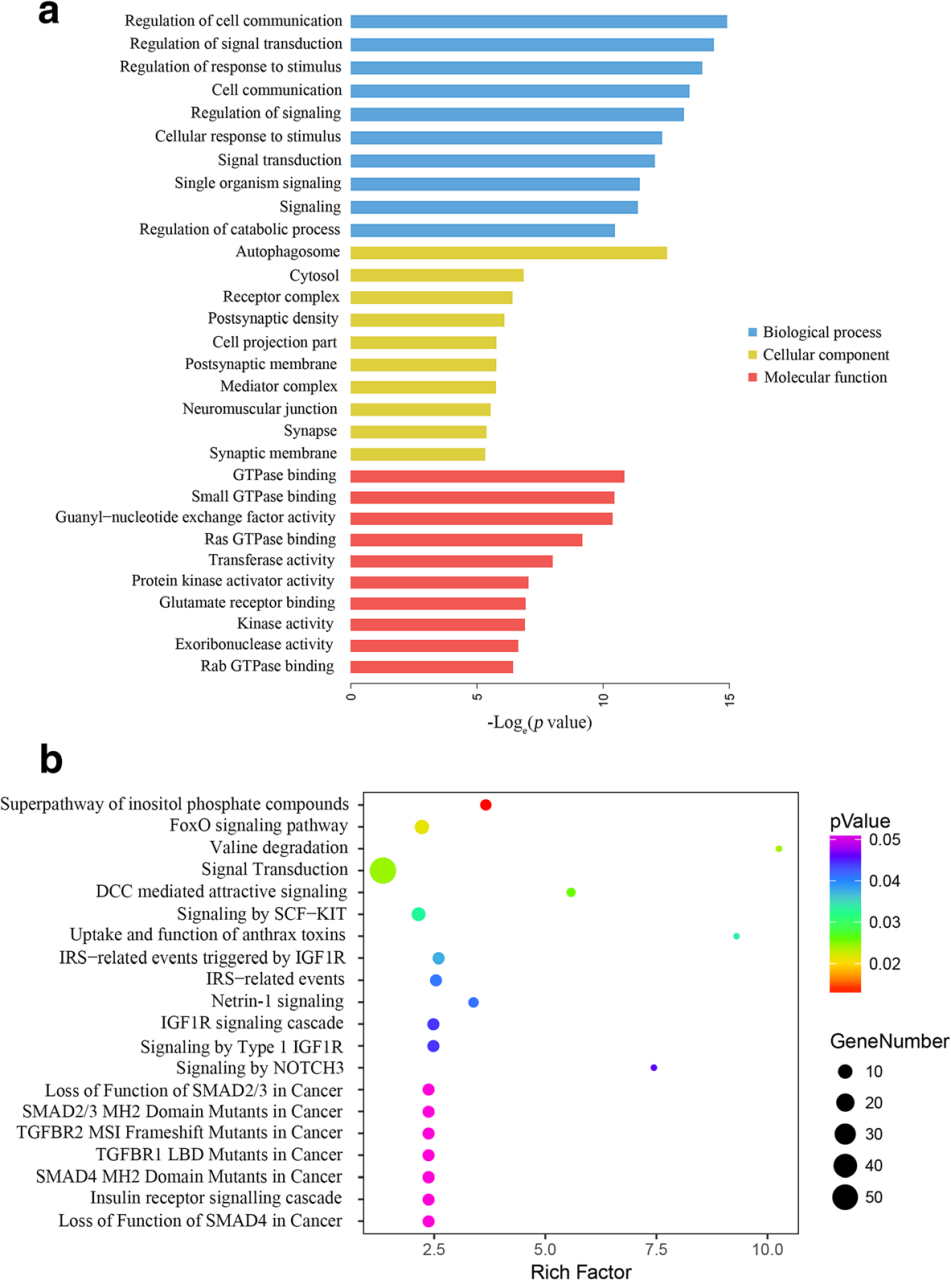
GO and KEGG analyses of the host genes of differentially expressed circRNAs. **a** GO annotations of the host genes of differentially expressed circRNAs. The bar plot presented the enrichment scores (−log_e_[*p* value]) of the top 10 significantly enriched GO terms in biological processes, cellular components and molecular functions. **b** Bulb map of KEGG analysis for the host genes of differentially expressed circRNAs. Rich factor represented the enrichment degree of differentially expressed genes. Y axis showed the name of enriched pathways. The area of each node represented the number of the enriched host genes of differentially expressed circRNAs. The *p*-value was represented by a color scale. The statistical significance increased from purple (relatively lower significance) to orange (relatively higher significance)

GO and KEGG analyses of the host genes of differentially expressed circRNAs were conducted to predict circRNA functions and molecular interactions among genes. GO analyses covered three domains: biological process, cellular component and molecular function. The top 10 enriched GO terms in biological process, cellular component, and molecular function are shown in Fig. 3a. The most significantly enriched GO terms in the biological process, cellular component, and molecular function categories were regulation of cell communication, autophagosome, and GTPase binding, respectively. The results of KEGG pathway analysis are presented in Fig. 3b. The host genes of differentially expressed circRNAs were mainly associated with superpathway of inositol phosphate compounds, FoxO signaling pathway, valine degradation, etc. Notably, the host genes of differentially expressed circRNAs were associated with important CRC-related pathways, such as Deleted in Colorectal Carcinoma (DCC) mediated attractive signaling, Netrin-1 signaling, Loss of Function of SMAD2/3 in Cancer, SMAD2/3 MH2 Domain Mutants in Cancer, TGFBR1 LBD Mutants in Cancer, SMAD4 MH2 Domain Mutants in Cancer, and Loss of Function of SMAD4 in Cancer.

### Analyses of circRNA-miRNA interaction in CRC

**Fig. 4:**
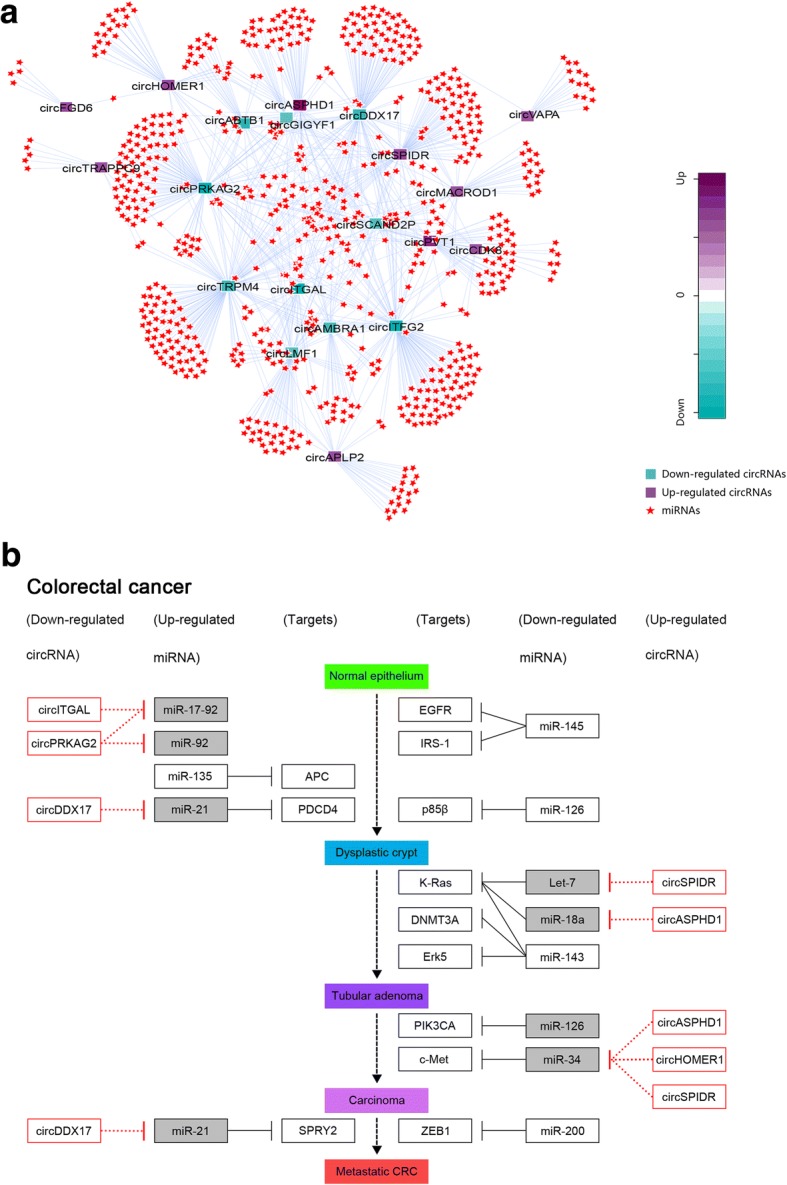
CircRNA-miRNA interactions are potentially involved in the initiation and progression of CRC. **a** Network of circRNA-miRNA interactions potentially associated with the KEGG pathway of COLORECTAL CANCER. The network was based on the top 10 dysregulated circRNAs in CRC and their predicted target miRNAs with experimentally supported or in silico predicted target genes which were involved in the KEGG pathway of COLORECTAL CANCER. miRanda software was used to predict the target miRNAs of circRNAs, and miRPath software was used to identify miRNAs associated with the COLORECTAL CANCER pathway. The purple square node represented up-regulated circRNAs. The cyan square node represented down-regulated circRNAs. The red star node represented miRNAs associated with COLORECTAL CANCER pathway. The log_2_FC was represented by a color scale, increased from cyan (relatively lower log_2_FC) to purple (relatively higher log_2_FC). **b** The top 10 dysregulated circRNAs were potentially associated with the KEGG pathway of MicroRNAs in cancer (section of “Colorectal cancer”). Target miRNAs of top 10 dysregulated circRNAs were screened according to the KEGG pathway of MicroRNAs in cancer, and target miRNAs associated with the KEGG pathway of MicroRNAs in cancer were identified. Among the top 10 dysregulated circRNAs, circPRKAG2, circITGAL, circDDX17, circASPHD1, circSPIDR, and circHOMER1 could potentially bind to miRNAs involved in the KEGG pathway of MicroRNAs in cancer. Modified from the KEGG pathway map of MicroRNAs in cancer

Mounting studies have revealed that circRNAs can act as miRNA sponge molecules, which regulate the expression of genes by adsorbing miRNAs. Hence, we predicted the potential target miRNAs of differentially expressed circRNAs using miRanda software (v3.3a) (results for top 10 dysregulated circRNAs were presented in Additional file 4: Data 3). A circRNA-miRNA interaction network was constructed for the top 10 dysregulated circRNAs and their predicted binding miRNAs using Cytoscape software (Additional file 5: Figure S1). To further explore the functional roles of circRNA-miRNA interactions in CRC, we screened miRNAs associated with the KEGG pathway of COLORECTAL CANER using DIANA-miRPath v.3 platform (Reverse Search module; Additional file 6: Data 4). DIANA-miRPath v.3 is an online software suite designed to assess miRNAs regulating selected pathways based on the functions of experimentally supported miRNA target genes (using TarBase v7.0 method of miRPath software) or in silico predicted miRNA target genes (using microT-CDS v5.0 or TargetScan 6.2 method of miRPath software). In this study, three methods of miRPath software were combined to perform the analyses. Based on the analyses by combining the results from miRanda and miRPath softwares, a circRNA-miRNA interaction network potentially associated with the KEGG pathway of COLORECTAL CANCER was constructed using Cytoscape software (Fig. 4a). The network was based on the top 10 dysregulated circRNAs and target miRNAs with experimentally supported or in silico predicted target genes which were involved in the KEGG pathway of COLORECTAL CANCER, and the network showed that most miRNAs targeted by the top 10 dysregulated circRNAs were associated with the KEGG pathway of COLORECTAL CANCER (Additional file 4: Data 3). Furthermore, we also screened target miRNAs of top 10 dysregulated circRNAs according to the KEGG pathway of MicroRNAs in cancer, and target miRNAs associated with MicroRNAs in cancer pathway were listed in the Additional file 4: Data 3. In this study, we regarded up- or down-regulated circRNAs as potential oncogenes or tumor suppressors, respectively, and so it was with miRNAs in MicroRNAs in cancer pathway. Up-regulated (or down-regulated) circRNAs targeting down-regulated (or up-regulated) miRNAs in MicroRNAs in cancer pathway were identified as potential functional circRNAs associated with MicroRNAs in cancer pathway. Consequently, we found that among the top 10 dysregulated circRNAs, circPRKAG2, circITGAL, circDDX17, circASPHD1, circSPIDR, and circHOMER1 could potentially bind to miRNAs involved in the KEGG pathway of MicroRNAs in cancer (Fig. 4b). In summary, the above results collectively indicated that the top 10 dysregulated circRNAs might play important functional roles in the initiation and progression of CRC by interacting with miRNAs involved in the KEGG pathway of COLORECTAL CANCER or MicroRNAs in cancer.

### circDDX17 is significantly down-regulated in CRC and correlated with CRC progression

**Fig. 5:**
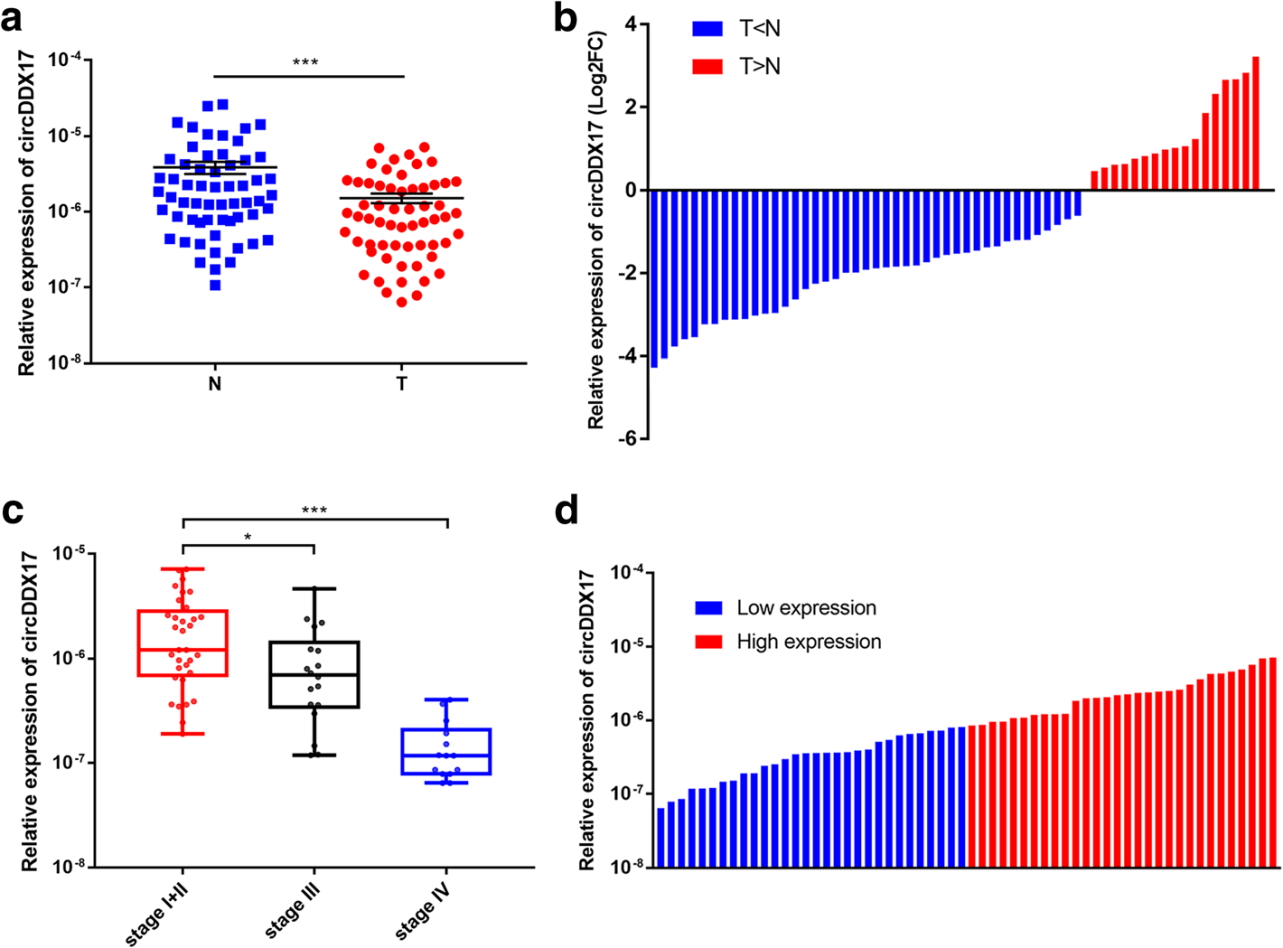
Relative expression of circDDX17 in 60 pairs of CRC tissues. **a** Expression levels of circDDX17 were decreased in CRC tissues compared with adjacent normal mucosa tissues. **b** 71.67% (43/60) CRC patients presented decreased expression of circDDX17. The bar chart presented the log_2_FC. **c** Expression levels of circDDX17 decreased with advanced TNM stages. **d** According to the median expression level of circDDX17, the 60 CRC patients were divided into two groups. The blue bars represented the low expression group, and the red bars represented the high expression group. Data are shown as means ± s.d. of at least three independent experiments. *p < 0.05, ***p < 0.001. T, CRC tumor tissues; N, adjacent normal mucosa tissues

As shown in Fig. 2a, circDDX17 was the most significantly differential expressed circRNA in qRT-PCR validation, and bioinformatic analyses indicated that it could potentially bind to hsa-miR-21-5p, which was involved both in the KEGG pathways of COLORECTAL CANCER and MicroRNAs in cancer (Additional file 4: Data 3; Fig. 4b). Hence, to further explore the functional roles of circRNA in CRC, we selected circDDX17 as a candidate circRNA for further investigation. CircDDX17 (hsa_circ_0002211) is derived from the DDX17 gene exon 2–8, with a spliced mature sequence length of 927 bp. We tested circDDX17 expression in 60 pairs of CRC tissues by qRT-PCR. The results showed that the expression of circDDX17 was significantly down-regulated in CRC tissues (Fig. 5a). 71.67% (43/60) of the 60 CRC patients showed decreased expression of circDDX17 in tumor tissues compared with matched adjacent normal mucosa tissues (Fig. 5b). Notably, we found that the expression of circDDX17 showed a decreased trend with advanced TNM stages(Fig. 5c). The expression level of circDDX17 in Stage III and IV patients was significantly lower than that of Stage I and II patients. In our study, stage I and II cases were grouped together due to the limited number of Stage I cases. Based on the qRT-PCR results, all the 60 patients were divided into high expression group and low expression group according to the median expression level (Fig. 5d). Statistical analyses showed that decreased expression of circDDX17 was significantly associated with lymphovascular invasion (*p* = 0.011), Depth of invasion (*p* = 0.007), lymph node metastasis (*p* = 0.020), distant metastasis (*p* = 0.012), and advanced TNM stage (*p* = 0.024) (Table 2). These results indicated that circDDX17 might play functional roles in the progression of CRC.

**Table 2:** Correlations between circDDX17 expression and clinicopathological parameters in CRC patients

Clinicopathological	Total (*n* = 60)	CircDDX17 expression^a^		*p* value
parameters		Low (%)	High (%)	
Gender				
Male	35	20(57.1%)	15(42.9%)	0.315
Female	25	11(44.0%)	14(56.0%)	
Age(years)				
≤ 65	26	12(46.2%)	14(53.8%)	0.455
> 65	34	19(55.9%)	15(44.1%)	
Tumor site				
Colon	37	17(45.9%)	20(54.1%)	0.426
Rectum	23	13(56.5%)	10(43.5%)	
Tumor size(cm)				
≤ 5	36	17(47.2%)	19(52.8%)	0.598
> 5	24	13(54.2%)	11(45.8%)	
Lymphovascular invasion				
Absent	22	7(31.8%)	15 (68.2%)	0.011*
Present	38	25(65.8%)	13(34.2%)	
Differentiation				
Well-moderate	42	19(45.2%)	23(54.8%)	0.464
Poor	18	10(55.6%)	8(44.4%)	
Depth of invasion				
T1-T2	21	7(33.3%)	14(66.7%)	0.007**
T3-T4	39	27(69.2%)	12(30.8%)	
Lymph node metastasis				
N0	27	9(33.3%)	18(66.7%)	0.020*
N1-N2	33	21(63.6%)	12(36.4%)	
Distant metastasis				
M0	43	15(34.9%)	28(65.1%)	0.012*
M1	17	12(70.6%)	5(29.4%)	
TNM stage				
I-II	26	10(38.5%)	16 (61.5%)	0.024*
III-IV	34	23(67.6%)	11(32.4%)	

*p < 0.05

**p < 0.01

^a^Using median expression level of circDDX17 as cutoff

### Silencing of circDDX17 promotes CRC cell proliferation, migration, invasion, and inhibits apoptosis in vitro

**Fig. 6:**
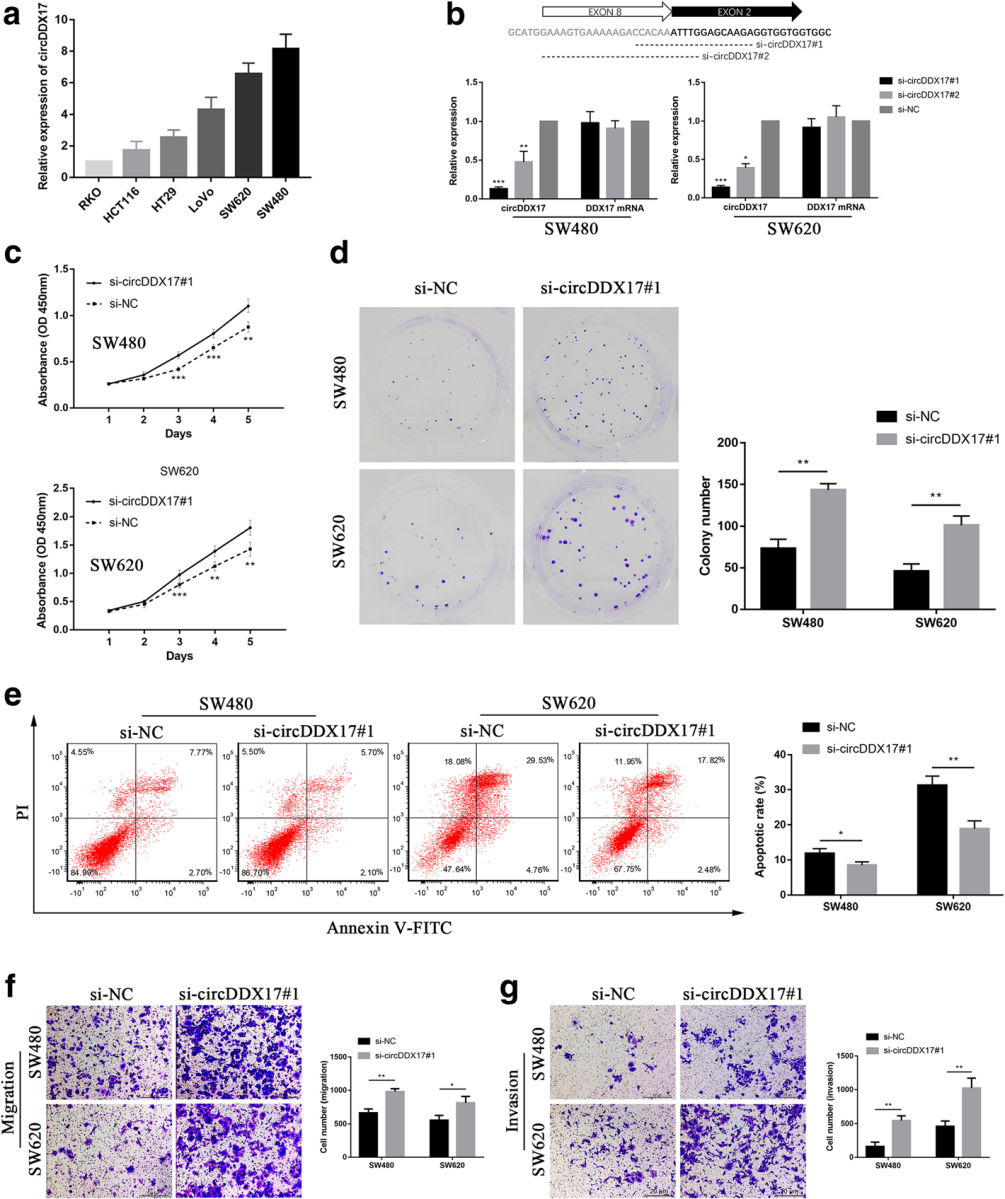
Silencing of circDDX17 promotes CRC cell proliferation, migration, invasion, and inhibits apoptosis. **a** Relative expression of circDDX17 in 6 CRC cell lines. **b** Schematic representation of the sequence around the back-splice junction site of circDDX17 and the siRNAs targeting the junction site (si-circDDX17#1 and si-circDDX17#2) (top). Results of qRT-PCR for circDDX17 and its linear isoform in SW480 and SW620 cells treated with siRNAs. **c** CCK-8 assay of SW480 and SW620 cells transfected with negative control siRNA (si-NC) or si-circDDX17#1 at the indicated days. **d** Colony formation assays of SW480 and SW620 cells transfected with si-NC or si-circDDX17#1. **e** Cell apoptosis assay by flow cytometry of SW480 and SW620 cells transfected with si-NC or si-circDDX17#1. **f, g** Transwell migration and invasion assays of SW480 and SW620 cells transfected with si-NC or si-circDDX17#1. Data are shown as means ± s.d. of at least three independent experiments. *p < 0.05, **p < 0.01, ***p < 0.001. Scale bar, 20 μm

In order to investigate the biological functions of circDDX17 in CRC, we silenced the circDDX17 expression in CRC cell lines. To choose the CRC cell lines used for silencing of circDDX17, we measured the expression level of circDDX17 in six CRC cell lines. As shown in Fig. 6a, RKO cells showed the lowest expression of circDDX17; SW480 cells showed the highest expression, while in SW620, derived from a metastasis of the same tumor from which the SW480 was derived, the expression level was moderately decreased. Therefore, SW480 and SW620 cells were selected for silencing of circDDX17. We designed two siRNAs targeting the back-splice junction sites of circDDX17 to silence circDDX17 expression. As shown in Fig. 6b, the siRNAs significantly decreased circDDX17 expression level, but had no effect on its liner isoform. We chose si-circDDX17#1 for the subsequent experiments due to its higher efficiency of interference. Cell proliferation was measured by the CCK-8 assay (Fig. 6c), and silencing of circDDX17 significantly promoted cell proliferation in both SW480 and SW620 cells. The colony formation assay showed that knockdown of circDDX17 significantly increased colony-forming ability of SW480 and SW620 cells (Fig. 6d). In cell apoptosis assay, the si-circDDX17#1 cells exhibited significantly decreased apoptotic rate when compared with cells of negative control (Fig. 6e). Moreover, in the transwell migration and invasion assays, knockdown of circDDX17 could significantly enhance the migration and invasion abilities of SW480 and SW620 cells (Fig. 6f and g). The above in vitro experiments collectively indicated that silencing of circDDX17 could promote the progression of CRC cells, which was in correspondence with the clinical findings.

## Discussion

CircRNAs were originally thought to be by-products of aberrant splicing with little functional potential [8]. Novel bioinformatic approaches, coupled with biochemical enrichment strategies and deep sequencing, have allowed comprehensive studies of circRNAs, and many studies have reported associations between circRNAs and human diseases, especially cancers [10–13]. However, the expression and function of circRNAs in CRC are still largely unknown. In this study, we screened the differentially expressed circRNAs between CRC and matched adjacent normal mucosa tissues by high-throughput sequencing. Consequently, we identified 394 up-regulated and 54 down-regulated circRNAs. Moreover, 15 circRNAs were identified as new circRNAs that had not been annotated in the circBase or circ2Traits database. 10 circRNAs were chosen for qRT-PCR validation in CRC tissues, and the results were highly consistent with the RNA-seq data, confirming the reliability of the RNA-Seq results.

CircRNAs are attracting more and more attention owing to not only their conserved expression among species but also their functions. So far, it has come to light that circRNAs function in multiple biological processes, such as miRNA binding, protein binding and regulation of transcription and post-transcription [8, 9]. Most circRNAs come from exons of protein coding genes via “back-splicing”. Thus, the forming process of such circRNAs could affect alternative splicing of the relevant pre-mRNAs, potentially leading to altered host gene expression [9]. In order to explore the potential functions of circRNAs differentially expressed in CRC, we performed GO and KEGG pathway analyses of the host genes of significantly dysregulated circRNAs. The results of GO analysis suggested the most significantly enriched GO terms in the biological process, cellular component, and molecular function categories were regulation of cell communication (related circRNAs and genes: hsa_circ_0006916 and HOMER1; hsa_circ_0002211 and DDX17), autophagosome (related circRNAs and genes: hsa_circ_0006508 and VMP1; hsa_circ_0009155 and AMBRA1), and GTPase binding (related circRNA and gene: hsa_circ_0000087 and USP33), respectively. The results of GO analysis showed that the functions of the circRNA host genes were associated with fundamental pathophysiologic processes essential to cancer development. Notably, the most significantly enriched GO terms in cellular component was autophagosome. The role of autophagy in cancer has been of particular interest, and research in this area has greatly expanded over the past few years [26, 27]. Studies have shown that the frameshift (FS) mutation of the key autophagic tumor suppressor, UVRAG, is expressed as a truncated UVRAG^FS^ in CRC with microsatellite instability (MSI), and promotes the progression of CRC [26]. In our study, hsa_circ_0006508, the circRNA derived from the newly discovered autophagy and CRC related gene VMP1 (vacuole membrane protein 1, enriched in autophagosome of GO terms) [28, 29], was significantly up-regulated in CRC tissues. Hence, hsa_circ_0006508 might be involved in the biological process of autophagy by regulating its host gene VMP1, and by which further impact the tumorigenesis of CRC. Moreover, the results of KEGG analysis revealed that the host genes of significantly dysregulated circRNAs were involved in many important CRC-related pathways such as Deleted in Colorectal Carcinoma (DCC) mediated attractive signaling, Netrin-1 signaling, Loss of Function of SMAD2/3 in Cancer, SMAD2/3 MH2 Domain Mutants in Cancer, TGFBR1 LBD Mutants in Cancer, SMAD4 MH2 Domain Mutants in Cancer, and Loss of Function of SMAD4 in Cancer [30–33]. In summary, GO and KEGG analyses suggested that the dysregulated circRNAs in CRC might be involved in the tumorigenesis of CRC. However, further research is warranted to confirm these findings.

In recent years, an increasing number of studies have shown that exonic circRNAs can act as miRNA sponge molecules [10–12], which regulate the expression of genes by adsorbing miRNAs. In our study, we predicted the potential miRNA targets of differentially expressed circRNAs using miRanda software, and constructed a circRNA-miRNA interaction network for the top 10 dysregulated circRNAs using Cytoscape software. More importantly, miRNAs associated with the KEGG pathway of COLORECTAL CANER were screened using DIANA-miRPath v.3 platform (Reverse Search module). Based on the analyses by combining the results from miRanda and miRPath softwares, a circRNA-miRNA interaction network potentially associated with the KEGG pathway of COLORECTAL CANCER was constructed using Cytoscape software. The network showed that most miRNAs targeted by the top 10 dysregulated circRNAs were associated with the KEGG pathway of COLORECTAL CANCER. Furthermore, we also screened target miRNAs of top 10 dysregulated circRNAs according to the KEGG pathway of MicroRNAs in cancer, and target miRNAs associated with MicroRNAs in cancer pathway were identified. Among the top 10 dysregulated circRNAs, we found that circPRKAG2, circITGAL, circDDX17, circASPHD1, circSPIDR, and circHOMER1 could potentially bind to miRNAs involved in the KEGG pathway of MicroRNAs in cancer. Conclusively, our studies indicated that the top 10 dysregulated circRNAs might play important functional roles in the initiation and progression of CRC by interacting with miRNAs involved in the KEGG pathway of COLORECTAL CANCER or MicroRNAs in cancer. Future studies should focus on these circRNAs and their related miRNAs and mRNAs.

In the last few years, many studies have demonstrated that circRNAs may serve as ideal biomarkers and therapeutic targets for cancer diagnosis and treatment [10, 34]. In this study, circDDX17 was the most significantly differential expressed circRNA in qRT-PCR validation, and bioinformatic analyses indicated that it could potentially bind to hsa-miR-21-5p, which was involved both in the KEGG pathways of COLORECTAL CANCER and MicroRNAs in cancer. Hence, in order to investigate the biological functions of circRNAs in CRC progression, we selected circDDX17 for further research. qRT-PCR results of 60 paired CRC tissues showed that the expression of circDDX17 was significantly down-regulated in CRC tumor tissues. After stratification by TNM stages, circDDX17 level showed a decreased trend with advanced TNM stages. Decreased expression of circDDX17 was significantly associated with lymphovascular invasion, Depth of invasion, lymph node metastasis, distant metastasis, and advanced TNM stage. In vitro studies showed that knockdown of circDDX17 could promote CRC cell proliferation, migration, invasion, and inhibit apoptosis. Thus, our results demonstrated that circDDX17 could function as a tumor suppressor in CRC, and could serve as a potential biomarker and a therapeutic target for CRC diagnosis and treatment. According to our bioinformatic analyses, circDDX17 could potentially bind to hsa-miR-21-5p, a miRNA with oncogenic properties which was involved both in the KEGG pathways of COLORECTAL CANCER and MicroRNAs in cancer. Previous studies had suggested that hsa-miR-21-5p was implicated in CRC tumorigenesis and metastasis [35, 36]. Hence, circDDX17 might inhibit CRC progression by regulating hsa-miR-21-5p and its target mRNAs. However, the exact regulatory mechanism of circDDX17 in CRC is still unknown. Further functional and mechanistic studies of cricDDX17 are needed, which are being undertaken in our laboratory while we present this study.

## Conclusions

In summary, our study revealed the expression profiles and potential functions of circRNAs in CRC. By high-throughput sequencing and qRT-PCR validation, numerous circRNAs were found to be dysregulated in CRC tissues compared with adjacent normal mucosa tissues. Bioinformatic analyses indicated that these dysregulated circRNAs might play important functional roles in CRC tumorigenesis. The expression of circDDX17 was decreased in CRC tissues and associated with unfavorable clinicopathological parameters. CircDDX17 functions as a tumor suppressor in CRC and could serve as a potential biomarker and a therapeutic target for CRC diagnosis and treatment.

## Additional Files

Additional file 1: **Table S1.** Primers for quantitative real-time PCR. **Table S2.** siRNA oligonucleotides used in this study. (DOCX 30 kb)

Additional file 2: **Data 1.** Fifteen new circRNAs identified by RNA-seq in 4 pairs of CRC and adjacent normal mucosa tissues. (XLS 34 kb)

Additional file 3: **Data 2.** Sequence (5′ to 3′) of 15 new circRNAs detected by RNA-seq in 4 pairs of CRC and adjacent normal mucosa tissues. (DOCX 36 kb)

Additional file 4: **Data 3.** Analyses of circRNA-miRNA interaction in CRC. Analyses were performed by using miRanda v3.3a software, DIANA-miRPath v.3 platform (Reverse Search module) and KEGG pathways of COLORECTAL CANCER and MicroRNAs in cancer. (XLS 498 kb)

Additional file 5: **Figure S1.** Network of circRNA-miRNA interactions in CRC. The network was based on the top 10 dysregulated circRNAs in CRC and their predicted target miRNAs. The purple square node represented up-regulated circRNAs. The cyan square node represented down-regulated circRNAs. The star node represented miRNAs. The log_2_FC was represented by a color scale, increased from cyan (relatively lower log_2_FC) to purple (relatively higher log_2_FC). (TIF 989 kb)

Additional file 6: **Data 4.** Based on the functions of target genes, DIANA-miRPath v.3 platform was used to identify miRNAs associated with the KEGG pathway of COLORECTAL CANER. (XLS 232 kb)

## Abbreviations

bp: Base pairs
CircRNAs: Circular RNAs
CRC: Colorectal cancer
DCC: Deleted in Colorectal Carcinoma
FC: Fold change
FS: Frameshift
GO: Gene ontology
KEGG: Kyoto Encyclopedia of Genes and Genomes
miRNA: microRNA
MSI: Microsatellite instability
OD: Optical density
one-way ANOVA: One-way analysis of variance
qRT-PCR: Quantitative real-time polymerase chain reaction
RBPs: RNA binding proteins
RIN: RNA integrity number
RNA-seq: RNA sequencing
rRNA: Ribosomal RNA
siRNAs: Small interfering RNAs
top 10 dysregulated circRNAs: top 10 up- and down-regulated circRNAs
VMP1: Vacuole membrane protein 1

## References

[1] Bray F, Ferlay J, Soerjomataram I, Siegel RL, Torre LA, Jemal A. Global cancer statistics 2018: GLOBOCAN estimates of incidence and mortality worldwide for 36 cancers in 185 countries. CA Cancer J Clin. 2018;68:394–424. 10.3322/caac.21492 30207593

[2] Memczak S, Jens M, Elefsinioti A, Torti F, Krueger J, Rybak A (2013). Circular RNAs are a large class of animal RNAs with regulatory potency. Nature.

[3] Fan X, Zhang X, Wu X, Guo H, Hu Y, Tang F (2015). Single-cell RNA-seq transcriptome analysis of linear and circular RNAs in mouse preimplantation embryos. Genome Biol.

[4] Granados-Riveron JT, Aquino-Jarquin G (2016). The complexity of the translation ability of circRNAs. Biochim Biophys Acta.

[5] Barrett SP, Salzman J (2016). Circular RNAs: analysis, expression and potential functions. Development.

[6] Guo JU, Agarwal V, Guo H, Bartel DP (2014). Expanded identification and characterization of mammalian circular RNAs. Genome Biol.

[7] Li Z, Huang C, Bao C, Chen L, Lin M, Wang X (2015). Exon-intron circular RNAs regulate transcription in the nucleus. Nat Struct Mol Biol.

[8] Han B, Chao J, Yao H (2018). Circular RNA and its mechanisms in disease: from the bench to the clinic. Pharmacol Ther.

[9] Liu J, Liu T, Wang X, He A (2017). Circles reshaping the RNA world: from waste to treasure. Mol Cancer.

[10] Chen B, Wei W, Huang X, Xie X, Kong Y, Dai D (2018). circEPSTI1 as a prognostic marker and mediator of triple-negative breast cancer progression. Theranostics.

[11] Wang R, Zhang S, Chen X, Li N, Li J, Jia R (2018). CircNT5E acts as a sponge of miR-422a to promote glioblastoma tumorigenesis. Cancer Res.

[12] Han D, Li J, Wang H, Su X, Hou J, Gu Y (2017). Circular RNA circMTO1 acts as the sponge of microRNA-9 to suppress hepatocellular carcinoma progression. Hepatology.

[13] Holdt LM, Stahringer A, Sass K, Pichler G, Kulak NA, Wilfert W (2016). Circular non-coding RNA ANRIL modulates ribosomal RNA maturation and atherosclerosis in humans. Nat Commun.

[14] Brown J, Pirrung M, McCue LA. FQC dashboard: integrates FastQC results into a web-based, interactive, and extensible FASTQ quality control tool. Bioinformatics. 2017;33:3137–9. 10.1093/bioinformatics/btx373 PMC5870778 28605449

[15] Dai M, Thompson RC, Maher C, Contreras-Galindo R, Kaplan MH, Markovitz DM (2010). NGSQC: cross-platform quality analysis pipeline for deep sequencing data. BMC Genomics.

[16] Kim D, Pertea G, Trapnell C, Pimentel H, Kelley R, Salzberg SL (2013). TopHat2: accurate alignment of transcriptomes in the presence of insertions, deletions and gene fusions. Genome Biol.

[17] Jeck WR, Sorrentino JA, Wang K, Slevin MK, Burd CE, Liu J (2013). Circular RNAs are abundant, conserved, and associated with ALU repeats. RNA.

[18] Zhang X-O, Wang H-B, Zhang Y, Lu X, Chen L-L, Yang L (2014). Complementary sequence-mediated exon circularization. Cell.

[19] Ritchie ME, Phipson B, Wu D, Hu Y, Law CW, Shi W (2015). Limma powers differential expression analyses for RNA-sequencing and microarray studies. Nucleic Acids Res.

[20] Xie C, Mao X, Huang J, Ding Y, Wu J, Dong S (2011). KOBAS 2.0: a web server for annotation and identification of enriched pathways and diseases. Nucleic Acids Res.

[21] Enright AJ, John B, Gaul U, Tuschl T, Sander C, Marks DS (2003). MicroRNA targets in drosophila. Genome Biol.

[22] Vlachos IS, Zagganas K, Paraskevopoulou MD, Georgakilas G, Karagkouni D, Vergoulis T (2015). DIANA-miRPath v3.0: deciphering microRNA function with experimental support. Nucleic Acids Res.

[23] Su G, Morris JH, Demchak B, Bader GD (2014). Biological network exploration with Cytoscape 3. Curr Protoc Bioinformatics.

[24] Glažar P, Papavasileiou P, Rajewsky N (2014). circBase: a database for circular RNAs. RNA.

[25] Ghosal S, Das S, Sen R, Basak P, Chakrabarti J (2013). Circ2Traits: a comprehensive database for circular RNA potentially associated with disease and traits. Front Genet.

[26] He S, Zhao Z, Yang Y, O’Connell D, Zhang X, Oh S (2015). Truncating mutation in the autophagy gene UVRAG confers oncogenic properties and chemosensitivity in colorectal cancers. Nat Commun.

[27] Zhong Z, Sanchez-Lopez E, Karin M (2016). Autophagy, inflammation, and immunity: a troika governing cancer and its treatment. Cell.

[28] Zhao YG, Chen Y, Miao G, Zhao H, Qu W, Li D (2017). The ER-localized transmembrane protein EPG-3/VMP1 regulates SERCA activity to control ER-isolation membrane contacts for autophagosome formation. Mol Cell.

[29] Guo X-Z, Ye X-L, Xiao W-Z, Wei X-N, You Q-H, Che X-H (2015). Downregulation of VMP1 confers aggressive properties to colorectal cancer. Oncol Rep.

[30] Castets M, Broutier L, Molin Y, Brevet M, Chazot G, Gadot N (2011). DCC constrains tumour progression via its dependence receptor activity. Nature.

[31] Fleming NI, Jorissen RN, Mouradov D, Christie M, Sakthianandeswaren A, Palmieri M (2013). SMAD2, SMAD3 and SMAD4 mutations in colorectal cancer. Cancer Res.

[32] Mazelin L, Bernet A, Bonod-Bidaud C, Pays L, Arnaud S, Gespach C (2004). Netrin-1 controls colorectal tumorigenesis by regulating apoptosis. Nature.

[33] Valle L, Serena-Acedo T, Liyanarachchi S, Hampel H, Comeras I, Li Z (2008). Germline allele-specific expression of TGFBR1 confers an increased risk of colorectal cancer. Science.

[34] Chen J, Li Y, Zheng Q, Bao C, He J, Chen B (2017). Circular RNA profile identifies circPVT1 as a proliferative factor and prognostic marker in gastric cancer. Cancer Lett.

[35] Lin P-L, Wu D-W, Huang C-C, He T-Y, Chou M-C, Sheu G-T (2014). MicroRNA-21 promotes tumour malignancy via increased nuclear translocation of β-catenin and predicts poor outcome in APC-mutated but not in APC-wild-type colorectal cancer. Carcinogenesis.

[36] Yu Y, Nangia-Makker P, Farhana L, G Rajendra S, Levi E, Majumdar APN (2015). miR-21 and miR-145 cooperation in regulation of colon cancer stem cells. Mol Cancer.

