# Supplementary material for: RNA sequencing reveals the expression profiles of circRNA and indicates that circDDX17 acts as a tumor suppressor in colorectal cancer
Source: J Exp Clin Cancer Res. 2018 Dec 27;37:325. doi: 10.1186/s13046-018-1006-x (PMC6307166; doi:10.1186/s13046-018-1006-x)
Supplement: Supplementary file 1 — Table S1. Primers for quantitative real-time PCR. Table S2. siRNA oligonucleotides used in this study. (DOCX 30 kb) [file 13046_2018_1006_MOESM1_ESM.docx]

**Table S1 Primers for quantitative real-time PCR**

| Primer ID | Sequence (5’ to 3’) | Notes |
| --- | --- | --- |
| circASPHD1-Forward | CTCCCGACTACTTCCTTCGC |  |
| circASPHD1-Reverse | TGCCAGAGGTCCACGATGAA |  |
| circHOMER1-Forward | CTCAGAGCCAAGGGCTGAAC |  |
| circHOMER1-Reverse | GGGTCAATTTGGAAGACATGAGC |  |
| circAPLP2-Forward | TGCCGGAGGGACAAAAAGCA |  |
| circAPLP2-Reverse | TGAGGCTCAGCAACAGCAAAT |  |
| circVAPA-Forward | TGGATTCCAAATTGAGATGCGTATT |  |
| circVAPA-Reverse | CACTTTTCTATCCGATGGATTTCGC |  |
| circTRAPPC9-Forward | ACGGAGCATGGAAGCATCAG |  |
| circTRAPPC9-Reverse | TCTTAGCACGTCCGATCTCAGT |  |
| circPRKAG2-Forward | CGGGGGCATCAGGTTTTTCT |  |
| circPRKAG2-Reverse | GGCTGTCCACCCTTTCGAG |  |
| circITFG2-Forward | AGACAAGCTGCTGTGGTCAG |  |
| circITFG2-Reverse | GCTGCCCTGTCAGATGTTCA |  |
| circDDX17-Forward | TGCCAACCACAACATCCTCCA |  |
| circDDX17-Reverse | CGCTCCCCAGGATTACCAAAT |  |
| circTRPM4-Forward | CCTAGTGGCTCTCACCTGCT |  |
| circTRPM4-Reverse | CCATGAAGATGGTCACCGGC |  |
| circLMF1-Forward | GACTGGGCATCTCGTCTTTCG |  |
| circLMF1-Reverse | CACCGATGAGCTGCTTGTTC |  |
| DDX17-Forward | GGTAAGGGTGGTCGTTCTCG | for linear transcript of DDX17 |
| DDX17-Reverse | ACGATCCCGATAGCTTGCAG |  |
| 18S rRNA-Forward | AAACGGCTACCACATCCA | internal control for 2^−ΔΔCT^ method |
| 18S rRNA-Reverse | CACCAGACTTGCCCCTCCA |  |

**Table S2** **siRNA oligonucleotides used in this study**

| Name | Target Sequence (5’ to 3’) | Notes |
| --- | --- | --- |
| si-circDDX17#1 | CCACAAATTTGGAGCAAGA | target DDX17 exon 8/2 back-splice junction |
| si-circDDX17#2 | GAAAAAGACCACAAATTTG |  |
| si-NC | TTCTCCGAACGTGTCACGT | negative control |
