# Supplementary material for: RNA sequencing reveals the expression profiles of circRNA and indicates that circDDX17 acts as a tumor suppressor in colorectal cancer
Source: J Exp Clin Cancer Res. 2018 Dec 27;37:325. doi: 10.1186/s13046-018-1006-x (PMC6307166; doi:10.1186/s13046-018-1006-x)
Supplement: Supplementary file 3 — Data 2. Sequence (5′ to 3′) of 15 new circRNAs detected by RNA-seq in 4 pairs of CRC and adjacent normal mucosa tissues. (DOCX 36 kb) [file 13046_2018_1006_MOESM3_ESM.docx]

**Data 2 name: sequence (5’ to 3’) of 15 new circRNAs detected by RNA sequencing in 4 pairs of colorectal cancer and** **adjacent normal mucosa tissues.**

>CBT15_circR_3863

AATCAATAATTACACACCTGACATGAACCGTGAGGATGTTGACTACGCAATCCGGAAAGCTTTCCAAGTATGGAGTAATGTTACCCCCTTGAAATTCAGCAAGATTAACACAGGCATGGCTGACATTTTGGTGGTTTTTGCCCGTGGAGGCTCATGGAGACTTCCATGCTTTTGATGGCAAAGGTGGAATCCTAGCCCATGCTTTTGGACCTGGATCTGGCATTGGAGGGGATGCACATTTCGATGAGGACGAATTCTGGACTACACATTCAGGAGG

>CBT15_circR_9581

GGGTTATTTTAGGGACACAGTGATGTACTATGGCTTTTACACCAATTCCACCATCCAGCACGGGAACAGCGGGGCATCCTACAACATGCAGCTGGCCTACATCTTCACAATCGGAGCATGCTTGACCACCTGCTTCTTCAGTTTGCTGTTCAGGCATGGCCAAGTATTTCCGGAACAACTTCATTAATCCCCACATTTACTCCGGAGGGATCACCAAGCTGATCTTTTGCTGGGACTTCACTGTCACTCATGAAAAAGCTGTGAAGCTAAAACAGAAGAATCTTAGCACTGAGATAAGG

>CBT15_circR_4008

GTCAAGGTGATTCTGGATAAATACTACTTCCTCTGCGGGCAGCCTCTCCACTTCATCCCGAGGAAGCAGCTGTGTGACGGAGAGCTGGACTGTCCCTTGGGGGAGGACGAGGAGCACTGTGTCAAGAGCTTCCCCGAAGGGCCTGCAGTGGCAGGTCCGCCTCTCCAAGGACCGATCCACACTGCAGGTGCTGGACTCGGCCACAGGGAACTGGTTCTCTGCCTGTTTCGACAACTTCACAGAAGCTCTCGCTGAGACAGCCTGTAGGCAGATGGGCTACAGCAG

>CBT15_circR_9672

CTTCTGACAAGGCTTCTAAAGAGCAACCACCCCGAGGACCTTCAGGCTGCAAACCGGTTAATCAAGAATTTGGTCAAGGAGGGAACAAGAAAAATCGGAGAAGGTGTCCAAGAGGGTCAGTGCGGTGGAGGAAGTGCGAAGCCATGTGAAGGTGCTGCAGGAGATGCTGAGCATGTACCGCAGGCCAGGGCAGGCCCCGCCCGACCAGGAGGCCCTGCAGG

>CBT15_circR_7520

GTGCCCTGGACCTTGGGGTGTGCTGGGCCATGCAGTGCTTTGGGGCTCTGGGGTACCCTGGGCTTTGGGGTGCCCTGGAACTGGGGTGCCCTGGGTCTTGGAGTATGCTGGGCCATGCAGTGCCCTGGGCTGTGGGATACTCTGGGCTTTGGGGTGCCCTGGACCTGGGGTACCCTGGGCTTTGAGG

>CBT15_circR_16907

GTTATTCCAGGATCTTTGGAGACCCGAGGAAAGCCGTGTTGACCAAAAGCAAGACAAATGACTCACAGAGAAAAAAGATGGCAGAACCAAGGGCAACTAAAGGCCGTCAGGTTCTGAACAGCTGGTAGATGGGCTGGCTTACTGAAGGACATGATTCAGACTGTCCCGGACCCAGCAGCTCATATCAAGG

>CBT15_circR_11935

TGGAGACCATCCTGGGCCTCACAGGAGCGACCATGGGAAGCCTCATCTGCTTCATCTGCCCGGCGCTGATCTACAAGAAAATCCACAAGAACGCACTTTCCTCCCAGGTGCGTGCTGCTCGTGGGGTGGCCTGGACGAGGGCTGATGGGTCTGGGCTGGGGAGAGGTGGAGGCGGGAGGAAGCCCCTGGTTCAGCGTTTTCAGATGGGTTCTATCAAGAAACTTAAAACCCACAAAAGGGACTTGGTCCAGTCAGCCTGGGGAACCCTGGGTTCACCAGGACGCACGGTCTTTGCCCAGCAGGGCCACACTAAGCTGCTCAAGCTGTGCATCGGGTTTTCCTGAGAGCCGACTTAGCTGAGTCGTAAGCGCTTTCCCCATGGTGTTTCTGCGATGCCTGATTGCCCATGGACCTGCTGCCACCTCTGAGTCCCCTCCCCAGCCGCCCACCCCTGAGGCCACCACCCACCGTTGTTCCTGGTGCGACCCTCCCACGGAGATG

>CBT15_circR_25101

GGCCACCGGGACCCAAAGGTGATCAGGGGGATGAAGGAAAGGAAGGCAGGCCTGGCATCCCTGGATTGCCTGGACTTCGAGGGTCTGCCCGGGGAGAGAGGTACCCCAGGATTGCCCGGGCCCAAGGGCGATGATGGGAAGCTGGGGGCCACAGGACCAATGGGCATGCGTGGGTTCAAAGG

>CBT15_circR_13588

GCCAGTTTGGCGCCGGCACGGAGTCCTACTTCTCCCTGCTGCGCTTCCTGCTCCTTCTTAACGTGCTGGCCTCTGTGCTCATGGCCTGCATGACGCTGCTGCCCACCTGGTTGGGAGGCGCTCCCCCAGGCCCTCCCGGCCCCGACATCTCCTCGCCCTGCGGCTCCTATAACCCCCACTCCCAGGGCCTGGTCACCTTTGCCACCCAGCTCTTCAACTTGCTCTCGGGTGAGGGGTTACCTGGAATGGTCCCCTCTCTTCTATGGCTTCTACCCGCCCCGCCCACGCCTGGCGGTCACCTACCTGTGCTGGGCCTTTGCCGTTGGCCTCATCTGCCTCCTGCTCATCCTGCATCGG

>CBT15_circR_12860

TGGTTCTGGGAGTTGGTGGATTCTGGCTTGGCCCCTGTGAGCCCCCTTGGTGGTGGTATCATCCGGAGAGGCTTCTCGGGTACCCCATTGCTGCCATCGCTGCCGAGCCGCCTTGTGTTGCCTGGAGAGGTCGAGCCCAGTACCCATGTGGTGTTCACCATTGAGGCTTAAGGGACAGAGAAAAGACAGCGTGGTCCCAGATAGG

>CBT15_circR_20124

CACAGGGAGCAGCGTAGGGAGCAGGCGTCCGTCGGCGAGGCCTTCCGAACGGTTGAGGATGCCGGCCAGTACCTGCACCAGAAGAGGAGCCTGATGGAGGAGCACGGTGCCACCCTGGAGGAGCTGCAGGAGCGTCTGGACCAGGCCGCCCTGGACGATCTCAGGACCCTGACCCTTTCGCTGTTTGAAAAGGCCACCGACGAGCTGCGGCGCCTGCAGAACTCAGCCATGACCCAGGAGCTGCTCAAGCGTGGGGTGCCCTGGCTCTTCCTGCAGCAGATCCTGGAGGAGCACGGCAAGGAGATGGCTGCACGGGCCGAGCAGCTGGAGGGGGAGGAGAGGGACAGGGACCAGGAGGGTGTCCAGAGCGTGAGGCAGAGACTGAAGGATGACGCTCCTGAGGCCGTGACAGAGGAGCAGGCAGAGCTGCGACGCTGGGAGCACCTGATCTTCATG

>CBT15_circR_7568

GTGTCCAATGTGAGGTGCAGCTGGTGGAGTCTGGGGGAGGCTTGGTACAGCCAGGGCGGTCCCTGAGACTCTCCTGTACAGCTTCTGGATTCACCTTTGGTGATTATGCTATGAGCTGGGTCCGCCAGGCTCCAGGGAAGGGGCTGGAGTGGGTAGGTTTCATTAGAAGCAAAGCTTATGGTGGGACAACAGAATACGCCGCGTCTGTGAAAGGCAGATTCACCATCTCAAGAGATGATTCCAAAAGCATCGCCTATCTGCAAATGAACAGCCTGAAAACCGAGGACACAGCCGTGTATTACTGTACTAGAGACACAGTGAGGGGAGGTCAGTGTGAGCCCAGACACAAACCTCCCTGCAGGGGCGCACAGAGCCACCAGGGGGCGCTAGGGACCGACTGAGTACGGGACAGGTCCCAGGAGCAGGTGCAGGGGGAGGTTTCCTTTTTCCTTGGCTGGAAAAGTCACCTTTATCTTCCCAGGACTCGAGCCTTCTAGGCTGTGATATTTTATTACTTGTATTTACTGTTCATTTATTATCATTAGTTTTTAAATTTTGGTAATTTTTACAACTCTATGGATATATTTTTAAGTGTATACTTTCAAGAAATAAACATTCCTAATTATTTGCACTGATTCTCCCAGAGTTTTATTAACATTTGTTGACATCAGCAACTACATAGCTATAGGGACAAACACTTTTAACGATAGACAGTTGTTTAGGCCTGAAACCCCGTTTATACTAAAAATTTACAAAAATTAGCCTGGCGTGGTGAGGGGCGCCTGTAATCCCAGCTACTCGGGAGGCTGAGCAAGGAGAATTGCTTGAATCCGGGAAGCGGAAGTCACAGTGAGTGGAGTGGACTGCCACTACACTCCAGCCTGGCGACAGAGCGAGACTTTGTATGAAAGAAAGAAAAGAAAAGGAAGAAAGGAAGAAAGGAAGGAAGGAAGGAAGGAAGGAAGGAAGGAAGGAAGGAAGGAAGGAAGGAAAAGAGAGAAAGAAAGAGAGAGGAAGGAAGGAAGGAAGGAAGGAAGGAAGGAAGGAAAAGAGAGAAAGAAAGAGAGAGGAAGGAAGGAAGGAAGAAAGGAAGGAAGGAAGAAAAGAAGGAAAGAAAGAAAAAAGATATATAAACACGCAGACCTATGCATATAACCATAGGGATTTATATTAAACATTACAATAAAATAATTCTAAAAATGTGTCCTAAGGAATCAAACATAATGATGAAGTAAATATAAATGTTAGAGTAATTTATAATTGATTTGTTATTTTTAATCGTTTACTTGAATTTATTTCTATTTGTTCATTTAAAAGTAGTATATTGGTCATTTCAAGAGAGCTAACAGTAAATTTCAGATGTTGTTGTTACGATATATGATAAGAATTTGAGGTGGTGAATGATAATTATCTTATTTCTCAATTATCTTAGTTATTCCATATTGTATTCACAAATCATAACATTGCTTCTTACCTTATAAATATAAACAACCATAATTTGTGAAATTACAATAATTTTCTTTATTTTAATTTTTTAATTTATCCCAGATCATTATCTTTTTCTTCGCTTCCAGATCTCACTGGATCATCTCGAGGGCCCATCCTCACCCCTGTCTCCCGAAGACTTCTGGAGAGGCTGCAGGACGGGCAGAAGGAGGAGCCCCGTGTGAGTCCACACGACCTGGAGCCTCCCTCTCCTTGGATTAGGCCATCTCCTCGGGATCACAGGGCTCTTCATTATCCTTACCCCGCTGTTGTACCAAACAAGCAACATCACACTTCAATTCATCAGGGTTTGCTTTAATTTTCTAAATCATCGTGAAGGTGATAATTTTAACAGTAACGTATCACAACCAAATAGGATAAGCCCTTTTCCATGGAACAGGGTTTCTTATCAGGATATACATGTATTATGGATTCTCAATTTATTTGTAGATGAGATGCTATTATCTCCATATTGTAGATGATTCTGCTAAGTCATCTCTTAAAATTAATTTTTCCAAAGACTCAAACAAATAAATGATATAATTACAAATTTCAGGTGTAATAGCTGAGCCAACATTGAGATTATATTAACATTTAGAACATGAACTTGCAAATATTGTTATTTTCCTGTCAGCTGTCCCCAATTGTGATTTTATACAGAATATTAGAAATTTATCCTGATAAATCAGGTTAAAATATTATATCACTAGTTTATTAACTTTTATAACTAATAATATAAAATGTTCCACATATTTTTTAGCCATGTTTTACTTACCCGTGATATGTGTATTTATTAATTTTTCATTTTAAGATGCCACCTTTATCTTTCTTATTTCTGGGATTTTATTCTAGTAGATAGAGCTGAATGCATTTTAATAATTATCATAATAATCACATTTACATTTTGCATTTTAATTTTCATATACATTTTATTAATATTTTAATTTCAATATAAAATATTTTCCTACAAATGTCAATTTTTGATTTTTATGAGATAAAATTAACCTATACAAAATACATATATTTTCAATGTACAGTTTGAGGGTTTCTGGCAAATGTGCACACATTTGTCTCCAGCATCTAAGTTATGATGAGGAGCAGGTCCATCTCCACAACAAGTGTCCTCTTCGGTGCTTCCAGTCAGCTCTCACATAAGGAATTTTTTTTCAAATTTAATATAGATACAGAGGGTAAATGTTTGGATTTGTCACGGGGGATTATTGAGTGATGCTGAGGTTTGGAATACAGATTCCACCATCCTCTCCCTCCACTCTCCAGCAGTCCACAGTGTCTATCATTCTCATAATTATGTCCATGTGTGCTCAATGCTGAGGTCTTACTTAGGAGAATATGTGGTATTCAGTTTTCTGCTCCTACATTAATTTGTTTAGGATTAAGGGCCCCAGCTTCTTTCATTTTACTGCAAAGGACATGATTTCATTCTTTTTCATGGCTGTGTAGTATTATACATTGTAGATGTACCGCATTTTGTATATTCAGTCTACCAATGATGTGCATCTGGTTTGATCTATGTCGTTGCCACTGTGAATAGCACAGCAATGAACATAGATGTGTATGTGTCTTTTTGGTAGAATTATTTGCTTACTTTTCAGTGTATACCCCGTGGTGGGACTGCTGCGTAAAATGATATCTCTGTTTTAAGTTCTTTGAGAAATCTCCAGTCTGATTTCCAAAATGGAAACACCAATTTATATTCCCCTCATCAGTGTATGTGTTCTCTTTTCTCCACAGTCCCAGCAGCATCCATTGTTTTTTGACTTTTTAGTGATAACCATTCTGAGTGGTGCATGGCTGCACACCTACAGTCATCTCATCTTTGATAAGGCTGATGAAAACAAGCAATGAGGAAGGGACTCCCTGTTCAATAAATGGTGCTGGGACAACTGGCTAGGCATATGATGAAGATTGAAGCTGGATATCTACTTTCAACATGTATAAAATTAACACAAAATTCATTAAAGTTTTAAATGTAAGACCTCAAACCATAAAAATCCTTGAAGACAACCTAGGAAATACTCTTCTTGACATCAGGTTTGACAAAAAAATGTTGGCTGAGTATCCAAAACCAATTGCAACAAAAAGAAAAATAGACAAGTGGGGCCTAATTAACTGAGGAGCTCCTGCTCAGCAAAACAAACAAAGAAGCAAACAAAACTAACAGCACAGTACTCAGACAACCTACAGAATGTGGAAAGATATTCACAAACGTTCCATCCAACAAAGCCGTAATATCCAGAATCTATAGGGAAATTAAACAAATCAAGAAGCAAAAAATAATAATAATAATAACCCCATTAAAAATGGGCTGATACGGTTTGCTGTGTCCCCACCCAAATATCAACTTGAATTGTATCTCCCAGAATTCCCATGTGTTGTGAGACGGACCCAGGGGGAGGTAATTGAACCATGGGGGCCTGTCTTTCCCGTGCTATTCTCTTGGTAGTGAATAAGTCTCACCAGATCTAATGAGACTTATCAGGGGTTTATCTGATGGGTTTATCAGGGGTTTCCGCTTTTGCTTCTTCCTCTTTTTCTCTTGCCATCACCAGGTAAGGAGTGCCTTTTACCTGCCACCGTGTTTTGGAGGCCTTCACAGCCATGTGGAACTGTAATTCCAATTAAACCTCTTTTTGTTCCCAGTTTTGGGTGTGTCTTTGTTAGCAGTTTGAAAATGGACTAATGCGTGGGCAAATGACACAGACACTTCTCAAAGGAATACATACAAGTGACCAGCAAATATATTTTTAAAATGTTTAACATCACTAATCATCAGAGAAATGTAAATAGAAAAATGTTCTGATTTCTGTCACTATAGGTCCATTTTCTTGTTTTGAATTTCATATACATGGAATAAAATATTATAGCTCATTTTTGTAAGAAGCTTTTACTATCTGTGAGGTTCATTCATGTGATAGCATCTATCAAGGTTTTGTCAACATATGTATAAATATGTATGTACTCATACACATATAGATATTTCATATCTGAATCAGTCCATTGCATTAATAAATGACAGATTATTAAATAAATCAGTTCATTAAGGGAATAAGTGACAATATGTATATCTATTTTCCTGTTGATGGAATTTAAATTTGTTTCCAATATAAATATCATAAACAAAACTGTCATACATATCTTTGTACAAGTTCTTCTGTTTATATTCACACATTTTTATTGATAAAATATGTTGAAATATAAGTATGCATTATACCTTTTCAGCTTTATGGAGCTATCACTGACAAATAAAATTTTCTGTATTTAAGGTACACCAATTGATGTATTGATATTCTTGGGGAAATGCTCATAATGATCAAGGTAATTGGCATGCCTATCATCTCAGAGAGTTAACATTTTATGCCTTTAATTTATTGTGTATGTGTGATGAAATCACCTAATATCTACTTGTCTGGCAAAAGATATGTTTATAATGCAACATTCATTAGTATAGTCACATTGCTGTAGGTTTGATCTCCAGAACTATTTCAACCTGTGTATTAGTCCATTCTCACCCTAATGTAAGGAACTACCTGAGACTGGGAAATTTATGAAGAAAAGTGGTTTAGTTGACTCACAGTTCTGCAGGCTTAACAGCAAGTATTACTAGGAGGTATCAGGAAACTTACAGTCATCACAGAAAGTGAAGGGGAAGCAAGGACCGCTTCACATGCTGGCAGGAGAGAGAGAAAGAGCAAGGGGAGATGCACCACCCTTTTAAACCTTGAGATCTTGTGAGAACTCTGTCACAAGAACAGCAAAAGGGAAGACCGCCCGCATGATCCAATCACTCCCCATCAGACACCTGCTACAACACTTGGGGATTAAAATTTGACATGAGATGTGGGTGAAAACACAGAGCCAAACCATATCATTCCACCCATGGTCTATCAGAAATCTCATGTCCTTCTCACATTGCAAAATATCATTATGCCTTCTCAACAGTCTTCCAGGCTTAACTCATTTCAGCATTATCACAAAAATCTATAGTCTAAAGTCACCTCTGAGACAAGGTAAATTTCTTCCCCCTATAAACCTGTAAAATTAAAAAGAAGTTAGTTATTTCCAAGACACAATGGAGGTGCATGTACTGGGTAAATGCTCCCATTCCAAATGGGTGTCATTGGCCACAGAAAAGGGGCTACAGGCCCCATGCAAGTCCAAACACCAGTAGGGCAGCCATTAAATGTTAAAGCTACAGCATAATTTCCTTTTACCCCATGTCTCACATCCAGGACACACTGATACAAGGGTTGGGCTCCCAAGGCCTTAGGAAGCTCCACCCTGTGGCTCAACGTGGTACAGTCCCCATGACTGCTTTGATAGGCTGGCATTGAGTTTCTATGGCTTTACAGGCACAGCATGCAAGCTGTTGGTGGATCTACCATTCCGGGGTCTGGAGGAAGGTGCCCCTCTTCTCACAGATCCACTAGGCATCTAGTGCCTAGTGCCCAGTGGGTACTCTGTGTGGGAGATCCAACACCACATTTTCCTTCCACACTGGCCTAGTAGAGGTACTCCATTAGGGCTCAGCCCTGCATAAGACTTCTGCCTGAATACCCAGACATTTTCATACGTCATCTGAAATCTAAGAGGAAGCTCCCAAACCTCAACTCTTGCCTTATGTGCACCCGCAGACTCAACACCACGTGGAAGCAACCAAAGCTTAGGGCTTGCACCCTCTGAAGCAATGGCCTGAGCTGGACCTTGGCCCCTTTAGCCATGGCTGGCAGGAGAGGGACAGGGATGTCCCAAGGCTGCACAGAGCAGTCGGGTCCTGGGCCTGGACCATGAAACCATTTTTCTCTACTGGGCTTCTGGGCCTGTGATGGGAGGGACCGCAGCAAAGATCTCTGAAATGCTCTCAAGATGTTTTTCCCATCGGGTTCCTCATTACTTATGCAAATTTCTGCAGCCCGCTTGAATTTCTTCCCAGGAAATGGATTCTTCTTGTCTACCACATGGTCAGGCTGCAATTTTTTTCAACCTTTTATGATCTGCTTCCCTTTTAAACATAAGTTCAAATTTCAGACCATCTCTTAATGAATGCATATGACTTACATTTTCAGAAACAGCCAGGGCAAATATTGAATGCTTTGCTACTTAGAAATTTTTTCTGCTAGATACCTTAAGTCATCTCTCCCTAGTTCAACATTGCACAGATCTCTAGGGCAGGGGCAAAATGCCACTAGTCTTTTTGCTAAAGCATAGCAAGTGTGAGCTTTACTCCAGTTCACAAGAAGTTCTTCATCTTAGCATCTGAGACCACCTCAGCCTGGACTTTATTGTCCATATCACTATCTGCATTTTGTTCAAAACCATTCAGTAAGCCTCTAGAAAGTTTCAAACTTTTCCACAGCTTCCTGTCTTCTTGTGAGCCCTCCAAGCTTTACCAGCCTCTGTCCATTACCCAGTTACAAAGTCACTTCCACAGTTTTAGGTATCTTAATAGCAGTGCCCACTCCTAGTGCAAATTTTCTGTATTAGTTGATTCTTACACTGCTGTAAAGAACTACCTGAGACTGGGTAATTTATGAAGAAAAGAGTTTAGTAGACTCACAGTTCTTCAGGCTAAACAGGAAGCATTACTGGGAGGCATCAAGACACACAATGATGGCCGAAGGTGAAGGGGATTCAAAAATCTTCTTTGCAAGATGGCAGGAGAGAGACAGCAAGGGAGACGGGAGGTGCCACACTTTTAAACCATCAGATCTCTTGAGAACTTTATCAGGAGAACAGCAAAGAGGAAGGCCACCCCATTACCCAATCACTTTATATTAGGCCCTTCCTTCAACATGTAGGGATTACAATTTGGCCTGAGATTTGAGTGGGAACACAGAGCCAAACTGTATCAACCTGCATAACTGAAAGTTATAACCTTTGACCAACATCAGCCAATTTTTTCCTCCTCCCAGCCCCTGGGAACTACTATTCTACTTTGCTTCCAAGAGCATGAATATTTTAGATTCTACATATAAATGAGTTCATGCAACATTTGACTCTCTGTGTCTCACTCCACTTAGCAAAGTGTTCTCTATGTGTTGTACATGTTAGAATTTCCTCGTTTTTAAAGGCAGAATAATATTCACTTTTAGGTAGGAATAAGCCACATTTTATCTGTTGATTCATAGATGGACATTGACCTATTTTCTATATCTAGGCTATTATGAATAATCTCACAATAAACATATATTTGTCACACTCACTTTATTTTCTCTAGATGTATACTCAGAAGTGGGTATATTCTATGTTCAATTCATTGAGTAATCTTGATGCTGTTTTTTCATAATGGCTGTACTAATTTGCATTTTGTTCCAAACCATACATGGATAACTTTGTACCACATATTCAGGTCTTTGTTTAAGTCTTAAATCCATTTTTAGCTGATTTATATGTATTGTGTGAGATAAGGTCCATTTTTTTTCTTCCGCATATGGATGCCCAGTTTTCACAGCACTTGTTGAAGAGACTGTTTTTTCTCTATTGTGTGTTCTTGGCAGTTCATCAAAGATCAGTTTATTGGGAAGAAATTGGTGGACTTCCAGATTGTCTGTAATGTTCTGTTGGATTCTATGTCTGTTTAAATGTCAGCATTATACTGTTTTGATTTACATAGATTTGATTTTGAAATTATAGAATATGATATATTCAGTTATATTTTACCCAAAATTATTTTGGCTATTTAAAGCTTTTGTATTTTTACATAAATTGGAGAACTTTTTAATATTTTTGTAAAACCATGCCATGGAGATTATATATTTATTTTATAGGCATATAATAGATATACCTAATTTATGGGAACATGTAATATATTGATGCATTTATAAACGTGTAAAGATCAGATCAGGATTGGTATATCTATCACATTAAACGTGTATATTTTCTTTATGCTAGGCACATTTGAACAACTCTCTTCTGGCTATTTTAACGTATACATTAGATTATCGTTAACTATAGTCACCTTACTCATCTATCAAACATTTAGGTTGTATTTCTCCTATATAACTGTATATCTGTCTTCAGTAATCATCCTCTCTTTATCCTCTTCTCTCTTGTATCCAACCAGGCTTCTGGTAAACAACAATCTACTCTCTGTCTTCAGGAAATCCAATGTTTTAGTTGTGACATAAAAGTAAGAAGATGCAATATTTGTCTTTCTGTGTTTGGTTTATTTAACTTAACATTATGGCTTCCACTTCCATTCATATTGTTGCAAATGACAAGATATCAACATTTATGGCTGAATAATATTCTATTGCCTACATCGATTATATTTTTATATCCATTTCTCCACCTACAGACACTGAGGTTGCTTTCATATCTGGGCTATTGTGAATAGAGCTGCAAGAACATGGAGTGCACATGCCTTTATGAGGCAGTGATTTTATCTTCTTTAGAACATACTCAGAATAGGATTTGCTGAGTCATCAGGTATTTCTATTTTTCTTTTATTTTGGAGTCTTCATACAACTTTGCACAATAGTGGAAATACAAACGGAAAAACCATTATAAAAAGCAGTTTCAGTTTTGAGGTATGATCCAAAAACACACAACATTTCATCATGATTCTCATTGGGAGTCTCTAATGAATCTGGTGGAAAGGCAGGAAGTTTTCTAACCTTGTTAAGAAAATTATGAGTTTGCACATTTTTCTTTTTGAGGCTGGAAATTGGCATGATTCAAGATGCGTAATGTTAGCAGGTTTCAGAATGATATAGCGAAAATCATAATGAGAAGGCAAGAGAGAGAATAAGAGAAAGACAGGAAAACAAAGAAACAAATAAATTTCACAAGAGAAGAAACTGCTGGATGTCAGTGTTGGGTTTTGTTCCTAGACATATCTGTACTGAGTGAGAAACCATGAAGCCAAGGGAAGAGTCTAGAACTTGTTCAAGTGAAGCATCAGCATATTTCTCCAAGGCACCTATTGTCACCCCATCACAAGGGTGATGAGTTTTTGAATACCATTAAATATGAGTTCCTAGTAAGTAATCATGTTTCCATGAAATTCAGTTAAAATGCAAAGGGTGTGTCCAGATCACTCATGCACAAAAATACAAAATTTGCCTTTGCATTTATGGCTGCATAGAGGTAAAGTCCATTGAGACTTCAGCAGGTTACAGAGATCTGTTAAGTTTTGGATTCCCATAGGAGAGTGCCTCTTAGTGAAGGTTGGACTATTAATTAAGTATGTACAAATTCCCTCTATGGGAGTAGTGTTCCATTTGTGTGGAATTTTTAATTCCTTATTGAATTGCAAAACAAATCCCAAGGACTGACTCCATGGAGTTTTACTGTAGTGTTCATAAAAATATTTAATAAGATTCCTTCCAGTGTTTCAAGGAAAGTTTTTCTCTAAAATTTTTTCTAATACATAAATTTTCCTGCCTAAAATTGAAACAGGCTGTTGTTAAAAAGACATACTGGAATGGCAGCTTTAATCTGTTGGTGATAGGAATGAGCATAATACATGAATCTCTTTGAGTGTTCTGCCTCATCCAAATACTGTATGGCAAAGGCTAGTATTGGAGGTAAAATCTCTAAATGTATTTGTTCCAGCCATCAAGTCATGGGGCAATAACAGATACATCCTTGAGGCAGAGGACCATAATGCCACAGTGCTAGTAGGAGTACATGTCGCCAAGGGAGTTCAAGGGTTCCTTAAAATTGTGATAATTTTAATGTAAGAACACCTTATTAGAGACAGGGTTTCAGCATGTTGACCAGGCTTGGTCTAGAACTCTTGACCTCTTGATCTGCCCGCCTTGGCCTCCCAAACTGCTGATATTACAGGAGTGAGTCACTGTGTCTAGCCAGTCCATTTATTTTCTTCTGCACATGGATACCCATAAACTTATGTGGAAGATTGTGGGAGGTTATAACAGTGTCATACTTTAGTATCAGCCATACCTCACAGAATCATTTTATAATAGTTCCTTTCATGGGTGTACTTCACTCAGTTCATGCAAAACCTGGCAGGTCACAGGCTAAAAACACAAAATACCGAAGTGTTTAACCAACTGAAAGGGCTGTAGCCTTTTGTCAATGAATTATTTCAAGACACGGAGAACACATATAGATTATAAATAAAATACTTTCACATCCCCTTAAGGGTGGAAATTGAGGAAATTTTACCTAAATGTGTCCAAAGGGCCCTGTGGCTTGGTTTCTGTCTAGGTCCCAATGATAGGGTTCTCCCAGGATTTTGTGGCTGTCTTGTTACACTTCATCAAGAATTAACCTCTGCTGTTTCCTCAAAGTGTTTAATTGGATAATGAATTTGTCTATAAATTGAAGAGTTGAAATACATCAAATATTAATTTGTAATAATCTGGCACAAATTATCTAAGCAAAGTCAATAACTAGATGTTTTTTCATTTATTTTTATTTAAAATCAGGACCTAAAGTTATGCTTTAATAACATCTGTGACCCTCTCAGCAGTTTTCTCTTCTGAGGATATGATCTGCTGTGGCAGTTTTCTTAGCTTCAATGTTACCTCTTTTTGCAATGACTACCGTCTTTATATTTGCCAGGAATCTGGGATAAAGGAGTGCTTCTAAGAGTTCCCTAACTTGCCCATTTTGGTGGGTGTTCCGGAACATATGAGATGCTCTGTTGTTAACAAAGCATCCCAAAGCCATGCACTGCCCTAAAATGTGTTTGTTTCCTAGTTTGACAAATTGGAAGTTCTAATAAATACAATCACTTCTGCCATCTGGGCTGATTTTACATCAGATAGAGGGCTGTATTCCAAAGAAAAGCTTACATTAGTAATAGCAATTTTAGTCAAAAGCCTAGAGTTTTATCATTGAGGTGCAATTCATAACAAATAATATTAGGTCGAGGTTCTCAGTGGCAGTGTCTAAATCTCTTAGGTGTACAGGGTCTTCCCTGTTAACATGAAGCATTTATAAGCACAGTCATAGTTTCCAGCCATGCTTCTCCCTGTCTCATTATCACCACAAACTATGGCCTCACCTGGAACTTGGGTTAATTTCCAAATAAGTAATTTTTTAGTCTTTATGCCTCTAGATTATTATGTGAGAAAGTTAACATTCAGTAGAAAGTTAAAAAGAACATTTGAACTGACTAAACAACACAGAAAATCAAGAATAAAATTCAAAGCCTAGATGTGAGAGGCTCCAGGCCTGGATAATGCAATAGTTCATGTAGGCAGGCAGTTTCTTTGCCCAGTTCTACACTGATACACCCAGAATGTCAGCTTCATGCCAGATTTGACTCCTATTATGTAGAGACATGGCAATACATTCTCAAGGGTCACATGAAATAATATGAAAATTGGTGGGAATAGGGGAGGAGACAACTCTGCAATTCTCATCTGAAGGACCAGGAAAGCCTGGACAGACCATCTCCCCAGCCTCCGTGACTGCACCACGTGCCCACATGGACACTCATCCCTGATAGGGTAAGAAGACTCCATTGATGGGGCTGAGCATTTTATGATAGAAATTACTAGAGACTGACGTGGAGGTTTCAACAACTAATATTTATAACCAAAATTTAATTACCCCCACATTGTTACCATTTTCTTCAGTGAAAAATTGCTTGCCATGATTAAGTTTTAAGTAGATTTCCAATGTTCACAACTGAGCTTCCAAGAGAGTCTTGAGAACAAAAACAATGAGGGCAGAGAAATCTATCTTTTCTGTATTCACCACTAAACTCAAGTGGACTCAGCACTGCCTTTGATCACTGCTGCTTCTCTGCAGAGTTCAGGTTTCTACTTCTCACAATTCTGACACACATTCTACCTCTCCTCAAATGTTTGGCCTCTGCTTCTTGTAAGGTCACCCTCTGTTCTTAACTTCTTCTCTGAGTCATTTTGTGAGGTGGTCATGAGCCATTAAATGGATATTTTATATTTTCCCAACATGAATCACATGAGTGGTCATGAATTATACTTCTGATTATGGCAGTTGATTTTTCTTGGCATGTTCATGACTAGTAATATTTGAAGCCATTTCATTCAAATCTTCGGGGCTTCGTTTTTGTTGCTATGACATTTTTTCTTCTATTGAGTCTTTCCACTAGTATTATAACATGACCTAGTATCCAGGCTCAGTTGTCATTAATAATAACCACATATGTCAAAAATCATGCATTCTTTTCACAGCAGACATAATTTCCTCTTTTCTGCAGATGAAGACACACTGCTGAGCTACCCCCACTTACAAGAATATATGCACAATTATGATATCTTCATTTATTTGACTAATAAGCTATATCATTCTCCCTTCAAATTCTTTACCCCCCAGAAGTCCTGGACAAATTTCTGCATCTGCTCAAACCATAAACTCAGAACTACATGGTGAGTAAAAGTCACCTGGTTCTGGATATTGGGTCCATCTCTTCCCCTCCAATGTCCCAGAGCACCTCAGCACACCTGTCCAGGTTCTATCAAGAAAGAGTAGCTCCTGCACACTGAAGGAAACAATTGAGTTAAGAGAGGACCTGCAGATGATAGACAATATTGAAAACTATTAATATGACAAAGGATTACTACCAAGCATGTGAAATAAGCTCAACGGGTGTGGTGGTTCATGTCTGTAGTACCAGCAATTTGGGAGGCAAGTTGCGCAGATCACCTGAGGTTAGGAGCTCGACACCAGCCTGACCAACATAAAGAACACCCTGTCTCTACTAAAAGTACAAAATTAGCTGGGCATGGTGGCATGCGCCTGTAATCCCAGCTACTCGGGAGGCTGAGGCAGGAGCATCACTTGAACCTGGGAGGTGGAGGTTGCGGTGAGCTGAGATGGCACCATTGCACTCCAGCCTGGGCAACAAGAGGGAAACTCCATCTCAAAAAAAAAAATTACAAAAAATTAGCTGAGCGTGGTGGTGGGCGCCTGTATACCCAGCTGCTAGGGAGACTGAGGCAGGAGAATCACTTGAACCCAGGAGGTGAAGGTTGCAGTGAGCTGAGATTGCGCCATTGCACTCCATCCTGGGCAACAAGAGTGAAACTCCATCTCAAAAAAAAAAAAAAAGAGCCTTGCAAAGGGCAAATAGATCATAGACAGACAGATAGATAGATAGACCTATTAGTATACATACATACATATATATACACTAATATTCAGGAAAATGCAAATTCATAATGAGATGTCTTTTCACCCTTCATCTCTGCTAGAAAGTTTGTTATCTGAAAAACAAATACATACATACATACTTATTAAAAGCTGGCCAGGATGCCTAGAAAGTAAAACTCATAGACCACTGGTGGAAATGTAAATTAGTGCAGCCATCAAGGGAAAAAAATAGAACTACCATATATTCCAGCAATCCAACTGCTAAGTATATATCTATTTAAATATTTAAAAGAAAAAACTAATATTGAAGAGATACCTGTACACCCATGTTTATTGCAGCACTAATCACAATTTCTAAGATATGAAATCAACATATGTGTCCATCAACAGATGAATGGATACATAAAATGTGATATATTTACACAATGGAATATTATTCAGCCTTAACAATGAAATTCTGCCATTTAAAGCAACATGGATGGAATGGGACACCACTATGTTGAGTGAAATGAGTCAGACACAGAAAAATAAATACCGCATTTCTCAGCGTTACTTCTAGAAGTAAATAGTAGAGTAGTGGTGATGAGATGCCAGGAATGAGAGAAGGCTGAGAGATAAGAAGAGGTTTGTTAACAAACACACAATTACAGGTAGACAGGAGGGATGTGCTCTAGTGTTCTACAGCACAGTAGGGTGACTACAGTTAACAATATATTGTACGTTTTCTGTTTACAAGAAGCCAGAAGAGAGAATTTTCTATGCTACCAACACAAATAAATGTTAGTGTCTGAACTGACGAATTTGCTCATTGTTCTGATTTTGGTCATACCAAGTGGCACACATGTATTCAAATATCACACTGTATCCCATAAACATAAGCAGTTATTATGTGCCAACTTTGAAAAATCCTTTAATTAAAAAGAATTATATTGGCGTACATTACAAATGATTCAACACAGAGACAGGAATAAATACCATTTTTCTTTGAAATAGTTAATTAACTAACAATGTAGTTACATTCATTTGCACCAAATCGTGTATTTGATAATGGTATGCATAGACAGAGTTATGCATAGGATAATATCTTTTAATTTTAGACTACTACTTAATACTATAAATATAAATAATTTTAAAACAACTAAGTAAAAAGAATAAAGCTGAGAAAATGTGTGTGTGGTGTGTGATGTGTGAGCTTTTTCTTGTGCACCACTGTGTCCTTGGTGGATGTGTGGTTCATGTGTTTGTTTTTATTTACTCTGTTTGGGGTTCTCTTTGCTTCTAGGATCTGTAGTTCAGTTTCTTTCACAAAATTGGGAACATTCTTCGCTATTATCTTTTTCAAATAGTTTCTGTGTATTTATAATTTCTCCTTCTCAGATTTAAAATATACACATACTATAATTTTGATATTAATGTTTAGTTTCTTTCTTCACTCTCTTTTCGTTTGCAATTTACTTTGTGAAATTTCTAGTGACATACTAATCACATGGTTTTATTGAAAAGCTGAGCCAGCTCTACTGAGGTGTGTGCCAAAAGATTGCTCGATGTTCATACAGCATTGCTTTTGATTTCTTACGCATTTCCATTTGATTTATTCTTAGTATTTTCATATTTCAGTTCCCTATCTATGTCCACGATTTCTTTAAGAGATTCTTGCATATGAATCATAGTTACTTTACATATCTTGTTTAATTAGATATTTATAATATCTGTTTCATCTACAAATCTCATGCTGATCATTTGTTTATTACGACTTTGGTACTTCTCATTAATGTATGTAATAATTGTTGATAGCCACAGATACTGGGATGGACAGTGGATACTGGCTTATTATTTCATTTTATGCATTTCTGCCTGTATTTGACCACACTTTACCTTTGCCAGGCCTTTACTGTGGAAGTATCTGTGAATCTTCTCAGAACTATATTTGACATTCACTTTTGCAGTGGACATCAAAGTTGAAGTCTGTTCTTCTGTGTCCACCAGAGACTTCAGATCCTCCAGTGATATCTTGTTTTTCTTTCCTGCTTGGCTTTGTCTCTTCACCTGTTCCCTCCTCCAGAGAATCATGTTCAGCTCCCTCAGGTGGATTTAAATGTTATTTAACTGACAATTGTGAAATTGGTGGAAAGCAATAGAATAAAGGGAGATTTTCTGACCTTTCCTGGGTTCATATTGTGAACATGAGTCTGGGTGTGACCTTCCCAATGTTTCTGAACTTCCTCCAGATGAGATGTTGGTCTGTGTGTTCTTGCTCTTTTCCCTGCTGTGGAGTCCTCTTGTTTTCCCCAGTTGTTCCCTCCCACAGCTCCAACGTTCTCTTTCGGTGTTATCACCTTACAGATTTGCTGACTAGAACTGCAGATTAAGGCTCTGATTAAATAAGAAGGAGGGGAGATACTTCTCAATGGAACTTAGGTGAAGACCTCTTTTCCCATCTCAGTTCCTAAGGGATTGCCCCAGTGCCCCTAAGATACTGGTTTTGGTGGCTTGCCCCTCCAGAATAATTTCTTTGTTCTCCAGTGGGGATATGGAAGGTGGGTCTGAACGCTTTTCAGAAGGGTGGGCACTTTTTCTCTCCTAGACAGACACAATGGGACAGAACAATTTTGGTGACTGTCCCCATTTTGGGGAAAAAGGATTCAATAGGATAGGAAAACTCTTCAGTCTGTGGTCCCTAAGAAATTCACCCTACAACACATTTACCACACTTGACTTCAAGCAATCCAATATATATGTGTGTTTTCATCTTGTAATAGCCTACATTTTACATGCCATACTCTGCCTCAGTTCAGCTCATACCCCAGCTTTGTTACTCTTTACAAGAACTTGCCTCTCCCTAGATTTCACATTTGCTGTTTATCTTAAAACTTCAAGTATCTAAAGTATTATTTTTAAAAAATGGCCAGTTGTGGTGGCTCACACCTGTAATCCCAACGCTTTGGGAGGCTGAGGTATGTGGATCACCTGAGGTCAGGAGTTTGAGACCACCCTGGCCAACATGGTAAAACCTGTCTCTACTAAAAATACAAAAAAAAAATAGCTTGGCATGGTGGCAGGCACCTGTAATCCCAGCTACTTGGGAGGCTGATACTGGAGAATAGCTTGAACCCAGGAGGCAGAGTTTGCAAGTCGTACCATTGCACTCCAGCCTGGGCGACAGAGTGAGACTCTGTCTCAAAAAAAAAAATTCCAAAATTCCAGCTCCTCTGTTTATCTATTTTTGTTGATACTGTTGTTGTAAAACATAAGTAAAATATATTATTCATCTATGTACATTTCCAAGCTGTGTAGAAGAATTTTTAATAAGACCCAGAGTAAAAAAAGAATGCAAATATGTAGGGGCCAGCCCTACAGGGTCTGTGGATCTTTCTCCCCATGTGCAGAGATGAGAGATCGTAGAAATAAAGGCACAAGACAAAGAGATAGAAGAAAAAACAGCCGGACCCAGGGGACCACTACCACCAAGACACAGACTAGAAGTGGCCCCGAATGCCTGGCTCCGCTGTTATTTATTGGATACAAGGCAAAAGGGGAAGGGTAAGGAGTGTGAGTCATCTGCAATGATTGATAAGGTCATGTGGGTCACGTGTCCACCAGACAGAGGGCACTTCCCTGTTTGGCAGCCGAGGCGGAGAGAGAGAGAGGACAGCTTAGGTCATTATTTATTCCATTCTCTTCTCAGAAAGATCAAAGACTTTAATACTTTCACTAATTCTGCTACTGCTATCTAGAGGGCGGAGCCAGGTGTACAGAGTGGAACATGAAAGTGAAACAGGAGTGTGACCGCTGAAGCACAGCATCACAGAGAGACGTTTAGGCCTCTGGAGGGCTGCGGGCAGGTTTGACTGATGTCAGGCCTTCCACAAGAGGTGGTGGAGCAGAGTCTTCTCTAACTCCCCCGGGGAAAGGGAGACTCCCTTTCCAGGTCTTCTAAGTAATGGGTAATGGGTGCCTTCCCAGGCACTGGCGCTACCGCTAGACCGAGGAGCCCTCTAGTGGCCCTGTCCGGGCGTGACAGAGGCTCACACTCCTGTCTTCTGGTCACTTCTCACTGTGTCCCTTCAGCTCCTATTGCTGTATGGCCTGGTTTTTCCTAGGTTATAATTGTAGAGCAAGGATTATTATAATGTTGGAATAAAGAGTAATGCTACAGACTGATGATTAATGATATTCATATATAATCATATCTATAACCTATTTCTAGTACAACTATTCTTATTTTACATATTCTCTTCATTACACTGGAACAGCTTGTGCCCTCAGTCTCTTGCCTCAGCACCTGGGTGGCTTGCCGCCCAGACAAATATTGTTAAGCTTCTTAATAGAAAAACAAATTATGGTAAATGTGTTCACTGGAATACTACCCGTCATTTATAATAAATTAATGCCTGATACACAGAGCAACAAGGTAAAATATCTAAGTATTTATGTTGAGTAAAATAAGCTAAACAAATAAGAATATATACTATGTAATTTCATTTTTATAAATTCTGATAAATAAAAATGCATCTGAAGTAAAATAATGAAGATAAGTAGTTGCCTGGGGTAATGGTAGAAGAAGGGAGGGGGAGAGGAGGAGGAATACAGCAGAACAAGGGGAAATGTTGAGAAGAATTCACTTGTCCACTTTCTTGGTAATGATAGCAGTTACATCATTTTTATTAGTTGTACATTTTAAATATGTGAAGTTTATTATCTTTCAATTAAGCCTCATAAAATGTCTTACAAGCAAACAAATGGAAACTTAGACAAGGAAAGAGTAATAGAAAGACAGAAAAAATAAGTTCAATGTCAGAAGTACCTGAAAATTAATGTGCCTGGATCCTAGTTCTCTCCATATTTTCAGAAGAGTGCTGGAGGGCAGCAAAACCACACATGCTCTTATTACGGAATGTGGGTTCTGATAAAAACACTAGACACATCCAGCTTTGTCCTGGAGTTGGTTTAGGGGGATGTCAGAGACAGTGATGAAGAGCACAGGGCCAGATACCGGGGTTCACTCATCCCAGACATGAGCTCCGAGACGCATACAGAGCCCCCCCATGTGTGGGTTTACTTCCACTTCTGTAAATGGAGAAAATATTGTCTCCTACAGAACATAGTTTACATGAATATTTAAAATGAAATAGGGTGATTAGTGCAAAGTGTTTATCACAGCACAATTTCATAATAAGACAGCATATTTTCCAAATGCAATCATTGCCAGCAAACTTCTACAGGGCACCGTCGTCTTATCTGGGTACAGCCTACTCCTCAAGGGTCCCACCCTAGAGCTTGCTATATAGTAGGAGATATGCAAATAGGGCCCTCCCTCTACTGATGAAAACCAACCCAACCCTGACCCTGCAGCTCTCAGAGAGGTGCCTTAGCCCTGGATTCCAAGGCATTTCCACTTGGTGATCAGCACTGAACACAGAGGACTCACCATGGAGTTGGGGCTGTGCTGGGTTTTCCTTGTTGCTATTTTAGAAGG

>CBT15_circR_1973

GAAAGGCCTTGGAAAGCAGTCGTTGCGCCAGACAGCCCAGGGAAGAGCGGCAGCCTGAGGACCTAGGGCCACCTGCTGTTCCCTGGGATTCATGTCCTTCTGGGGAGGAGGGAGGACCCAGGACAATGGCTGCTGTTCATGATCTGGAGATGGAGAGCATGAATCTGAATATGGGGAGAGAGATGAAAGAAGAGCTGGAGGAAGAGGAGAAAATGAGAGAGGATGGGGGAGGTAAAGATCGGGCCAAGAGTAAAAAGGTCCACAGGATTGTCTCAAAATGGATGCTGCCCGAAAAGTCCCGAGGAACATACTTGGAGAGAGCTAACTGCTTCCCGCCTCCCGTGTTCATCATCTCCATCAGCCTGGCCGAGGCTGGCAGTGTTTATTTACTATGCTGTGTGGAAGCCTCAGAAACAGTGGATCACGTTGGACACAGGCATCTTGGAGAGTCCCTTTATCTACAGTCCTGAGAAGAGGGAGGAAGCCTGGAGGTTTATCTCATACATGCTGGTACATGCTGGG

>CBT15_circR_7558

ATGTCCAGTGTGAGGTGCAGCTGGTGGAGTCTGGGGGAGGCTTGGTCCAGCCTGGGGGGTCCCTGAGACTCTCCTGTTCAGCCTCTGGATTCACCTTCAGTAGCTATGCTATGCACTGGGTCCGCCAGGCTCCAGGGAAGGGACTGGAATATGTTTCAGCTATTAGTAGTAATGGGGGTAGCACATACTACGCAGACTCCGTGAAGGGCAGATTCACCATCTCCAGAGACAATTCCAAGAACACGCTGTATCTTCAAATGAGCAGTCTGAGAGCTGAGGACACGGCTGTGTATTACTGTGTGAAAGACACAGTGAGGGGAAGTCAGTGTGAGCCCAGACACAAACCTCCCTGCAGGAACGCTGGAGAAAAACAGCTGCAGTGGGCGCTCAGGACCCACTGATCAGCGTCAACCTCAGAGGCAGGTGCACAAGGAGGCTGATTTCCTGTCACGATGTGGGACTTCATCTCTTTACAGTTTCTCTAGTGAACCTCTCTAAGTTCAGAACTCTGTGCTTACTAATGTCATCTGTACATATTTTTTAAAATGATTATTTTAATATGAAAACCTATTCTCCTGTGTACAAAATGCGGATTGATCCTTACAGAGGAGATGAAAACTCCTCAACCATGGTCACCATGATCAGAGTTCTGAGGAAACTAGGGGCTTCCTGGTGAGTCTTCTCCAATCAAATTCAGGACAGGAACCTCAGAAAGATTCCTGACTAGAACTGAGGTATTGCTCCTCACAAGAGAGCTCTAGCTCCGGGGGGTCTGTTCCTGCAGACCCCTGATTCGGTGGTGTATGAATAAAATGTACACTGACACACAGATACTCTGTTTTTCCAGTGCAGCTGAGGGTCCGAGGCCACTTACAGACTCCAAGAAGAATCCTGTAAAGAATGGCAGCAGTGGCCCTGAGCAGCTCGCACTCCAGGCGTGTATTTAGTATAGAATTAACAACAGAAGCTCTGAGTCAACACACTTGTGGATAATTAACATGGTTAAGAGAGTAGTTCTACGAATGAGTAAAGCTCAGGGACTGTGGTCTGAAGTAAATACCATTAGGGGGCAATATTCTTGGTCGACCTCCCGCTGAGAGGGTCTTCTGGCTTAAAGGTTAGTTAATGGGGTAGGGTAACCAGACTTAACTGGGGAAGCCTCTGTTGTCCCTAGTATTTACCCTACAACCTAATGCTCTAAGGTAAGAACCGGCCATCTTCAGCCTGTTCAGTTATTGCAAGCTATGTAAACTTCCGGCCTTCCAAAAAAGTTTGTGACTATTTCCTATAACTTTCGTTAATATTTCCCTTTAATATTTCTGCCACCATCCTGAATGAATCCCAGCAAGAACAGTCTTTGGGGATTCTGATTACAGAAAAAAGAGAGGCTGGGCCAGGGTCAGTGTCATGTAGAACCTCACAGCTTTCTTGTGGGACCCTTCTCCTGACACCGAAGTATGCAAATCAGCATCAGCACTGATCTGGTGCTTCTTTTGTTCCTGATTCATTTACTTTCTTTTTTAGTTGTTGTTCTCATTTTTCCATTTGCTTTTCCTGCTTTCTGAGAAACAAAGATGTTTTTGCTGTGGTCAAAATTCCAGGCCTCAAGCCCTTTTCCTGGAGCTCAGGTGGGTCTCAGGCTGTGGCTGCTGCAGTCACGTGGGAGAGGCTGGTGGGACTTTCTTCACTCCTCGTCACTCAGGACCCTCCACTGTGTTGCATGGAGACTCATGTCGAAATACAAGTTGCCAGTGAGAACACAGGGGACAAGCTTGTTTGGTTAACGTGGGATGTGGATGTGTTTCTAATCCTGTTCTGAAAAACCTTCACACAGTAACGTTCTTCACTAGTGGAGGAGGAAGTGGGTGTGAACGTTGTCAGAATAAAAATGGAGCCACGTGTGTTATAATCTTTACAGGTGAAGCTGGAGGAGGTCATGAATAGAGGGTTCTCATGCACACATCCCTGATAACAAGAACTACCCTAAAAATACTCTGCACAACCACAACCTTGAACAAAGGCTACCACAACAATAAGAGAATTAATACTGTGGGGATATCTGCCCTGCAACTCCCTGTTCAGCCTTAAACTGATTCTACCCTTGTTATTGATTCTTCTACCCCAGGATAATTGTCTCAAAATAGCTCATGTAATCTTCTCATTTATCCTTTAGAATACTTGTCTTCCTTTACCAACCTAAATGTGACCATGGATAATCCCATTGCAATGCTCATTTTCAAATAAATACGATTTGATTTTGGAGAATCTCTTTCTGTCTGATATTTAGGTTTGACAAGATGCAGACCACTTTCCTGTGAGATGTAGGGAATGACAATTTTTGGGGGTGGCTGGAAACATCCAATACTCTCAGGGCCGGCCATCAGTAAATGCAGGCTGGATGTCTTAGAAAGAGCTCAAGGTCCTTAATCACCATGGAGTTTTACCTTCTCCAGATCTGTTCTGATGGAATCAAGGCCAAGCTGGTTATCAAAGATAATCTACCTATCATAGAGTCAACTGATTACAGTTTTAATAAAGTCTATTAAAAATCCACACTACCACCTGGATTAGTGTTTGGTCAAATAACTACAAAGTATTGGCCAGCCAAGTACACCATAAGACAGACCATTACCCATGTAGAAAAACATTTAACATGAGTTCTAGGTTCTTATATTGTTAAAGGTGTAAAACCGATTATTTTTAAATTACGCTATTTTTATTTTATCGAGTTGTAGAAGTTTCATTTACGTTTTGGATGTTAACACTTCTTTAGATACATGGTATATTATCCAATTCTGTGAGTTGGAATTATTTCGTTGCTTTGCAGAATCCTTTTTTAAATCTAGTCCCACTTGTCCAATTCTGTTTTTTTTTTTATGTCCTTTGAATGTAAAAGCCAGAAAAAGATTGCTAATTTTTTGAGGGTTGGGAGTTTTACAATTGCAGGTATTTCATTTAAATATTGAATTCATTTAGAGTTAATTTTTTTTGTTTATTCTAACCTAAAATTCTTAATTCTTTGCATGTGAAAATCCAATTTTCATAACCTGCTCTTTGGAAGTCACTATAATTTAGCAATTGTATATCGATTGTTCTCATGCTGAAAATCAGCTGGCCATCAGTATGTGGGTTTATATCTCAGCCCTGTATGCATTTATGCCAATACCATTCTGATTTATTACTCTGTGTTTCTAATAAATGTTGAGGCCTGGAAGTGAAATACCTCAAGCTTTCTTCTTGCCTTGTTTCAGATATTAGACCAAAATATTCTAACCTTTTACTATTGAGTATAACAATAGCTGAGACTTTTCTTAATGGCTTTTATTATGTACAAGTTGTTTGCTTGTCTTCATACTTTGTTCAGAGTTTTTATAATGAAACTCTGAATTTTTTTTCAAATGTTTTTGTGTCTGATGAAATGTTACTGAGATATTTTTTCTTTAGTTTTTTAATGTGGTGTACCAAATTGATTGATTTGAGAATGTTGAATCATCTGTGCATCTCAGGAAGAAATTTGAGTTGGTCATGGTGTATGGTCTTCTATAAAATCTTTAGAATTTATTTTACTGTTGTTGGACGGTTAATTTATGTCTATTAATGATATTGATCTATGGTTTTATTTAATTGTGGTTCCTTTGTCTATTACTGGTAATACTGTAACGGTAGCCTCATAGAAAGAGTTTGGAGGGTTTGTTGCAGACTGCCTTTAAAATAGATTTTATCAGTGGAGAAACGGTGATAGTTTTTTTCTTCAGTTTTCTGTTGGGAATAGTTTTATGTATTTTAAAAGATATTTTGAATGACTTAACCTGCTTAAAGGGCTTACATAATATTCCTTTCTTCCATGCTTTTTAAAGAACTGCTTACCTTTCCTAATTATTTTTAGGGTGATTTTTTTCATAAAGATTGTGCAATACATTTTGAGGTGAAACTTAGTGGATTTTTTCTAATGAATTAGAAATAATAAATCACTTAATTGACTATTGTATTAAGGTTGATTTGTTGAATATTTGCTAAAGGCCAGTTCTTTAAGCTGTGCCATGTACTAAATCCCTAACCGACTATTTTATTCAGGTTGATTTGTTGAATATTTGCTGCAGACCAGTTATTTACGCTATGGCATGTAATACATCCCAAATGGCAGTAAGTCATTGTTTATTTAGCTTTTGTGCTTATATTTTTCAGAGGAAAAAACACTACTGTAAATTGTAAATAGCCAATACATAACAGTATTGTATGCAAATCTGTGACTGTTGGCAGTGTCATCTCTGAGAAACAGATAAAGTTTATTTACTATATATAAAAAAAAGAGTTTGGAAGGTGGACTCCTCACCAATTTTTGAAAGAGTTACGGAAGGGTTGCCATTACTTCTTTAAATGTTAAGACTCATTTTATGATTTAACATATAACCTATCACGGAGAATGTTCAATGGGTGCTTGAGAAGGATGTGTATTACATGGCTCTTGGTTGGAAGGTTCTGTAAATGTCTTTCAGGAAAATTTGTTCAACAGTGTTGTTCAAGTTCAGAGGCTTGTTAACAATTCTCTTTCTGAATTTGCTATACATTATTGTTAAGTGAGGTATTAAGGTTTTCCCATATTTTTATATTGTTTTCTATTTCTCTATATATGCATATATATATTAAAGTTTGCTTAATGCATTTATATTTGTATTTGTACACATGTGTAAAAAATAAAATAGTAATTGCCTAGTGAGTTTCATGGCACAGTCACATTATGAATAATCATATTTTCCCAAACGCTGCCATTCCACTAACTCCTCCAGATACATGGTATATTATCCAATTCTGTGAGCTGGAATTATTTCGTTGCTTTGCAGAATCTTTTTTTGTTAATCTAGTTCCACTTGTTCAATTCTGCTCCTCCAGGAGTCTCATATCTCCTCTGGGCACTGCCTTCTTCTCAGGCATCCCACACTGGAGCTTACTATAGACGAGGAGGCATGAAAACAGGACACTCCCTCTCCTGGTGAAAACAATCCCAGACCCAACCCTGCAGCTCTGGGAGAAGAGCCACAGCCCTGGGATTCCCAGGGGTTTCCATTTTGGTGATCAGGATTGAACACAGAGGACTCACTATGGGGTGTGAGTTAAGCTGATTTTTTCTTGTTTGTATTTAAAAAGGTGACTCATAGACAACTAGAGTGAGTGGATATGAGTCAGAGAAACAGTGGATATGTTCGGCAGTTTCTGACCAGGATGTCCATGCATTTTCAGGTGTTCAGTGTGAGGTGCAGCTGATTGAGTCCATAGAGGACCTGAGACAATCTGGGAAGTTCCTGAGACTCTCCTGTTTAGCCTCTGGATTCACCTTCAGTAGCTACTGAATGAGCTGGGTCAACAAGACTCCAGGGAAGGGGCTGGAGTGAGTAATAGATATAAAATATGATGGAAGTCAGATATAACATGCAGACTCTGTGAAGCGCAGATTCACCATCTCCAAAGACAATGCTAAGAACTCGCTGTATCTGCAAATGAACTGTCTGAGAACCTGAGGACATGGCCATGCATGGCTGTACATAAGGTTCCAAGTGAGGAAACATAGGTGTGAGTCCAGACACAAAATTTCCTGTGTCTGGAAATTTTGAAAGAAGAAAGAAGAAAGGAGTCTGGGCCGAAGGGGACACTCAGCACTCACAAAACGGGTGGAGGCCTAGGGCAGGTACAGAAAAGCAGTCAAGGGCTGCTGTCCTTCAGGATCTGTGCCTTCCTCTGCATATAGCAGGTGCCCTCGAATCCTCTGCACTTTTATGTTTCTGTGCCTACCATGAGGTCCCTGGATTACAAAACTTTAATTTGAAAGAGGAAACATTCTTATATGTCCCAAAAACCGATGTAAGTATTGGAGGTGTAAAAATGCACAGGCAGTTGGATGAGGCTGTAGACACTGCCAACCCACAATGCCAGTCTCACAACTAGCACTGGAGAATAGTGGGAGTTCAATGGAGCTTCCTACCTATCTTGTGGTCCAAGCTAACTCCAGCGAGGCCATTGGTGCCATCAGGGACCTCCCATATGTCCCAGCAGCAGCCATGCCTCAGTATCTCTATTGTGCACAGCCATAGTCTGGGAGGAGCTCCCAGGATGGGTGTCTTTGGCACACACAGGTGATGGGTGTTAGAGTGTAGTGCAGCAGCTGGCTGCCTGGTCTATTGGGCTCCCTGATGTTGGAGGGATTAGAGGTGGATTCTCAGGGCCAGCACACTGGATTTTTGTATGAAAACCATGATTTTACTTCATTTTCTCAGATGACATAGATAATTAATAACACAATCTGCAAACAATTGTAATTTTCAGCTTTAGCCCAAATTCATTGTTTCTGAATTCTGTGCAGGATCCAGACATGGTACTGCCCTTCTCATGAGAAATTGTTCGACCTAAACTGAAACCAGTTGTTTCTCGTATACTTTGGTTCTCCCCATGTGCAGAGATCTTGATTAGAGCAAGTTTGGTACTTTCCACGCACTCACCCTCACCTCCCCAGATAAAGAGCAGAAGTTCTCCTTAGACTGAGTCTGAGGGAGGAGCTGTTCCTGTACCACTCAGGGCCTGCGGAGACCCCCAGGTGCAGCTTCACTGAGTCAGGTGTTTCACTCCCTGTGATTGCTGCTCAGGTCTAATTGTGGCTCGGAATTAGGACAGTCTTCAGGTTATCACAGGTCAATCATATTCTAAAAATCATCGTTATCACACACCATGGTAACAATTCAAGGTTCATTTTTCTAACGGCAGTTTCTCTTTTTTATTTGGTTACAAGTTTGAGGAAAGGAACATCTGATAATACTTTTTAACCTAACCTCGAAATCTACTGAATTGTTCTAGGAGACTCACAAATTGGACAAAGTGAGCTCTTTATTCTCATAAAAATGTGTGGTTTTGGGAATTTCACTGTGTTGCCCAGAACCTGTTAACATCAACAACTATGTTTCTCAGCACACTTCTGGCTTGAGACGTCCTTGCAGACCCTCTCCCTCACTTGCACTGTCTCTGGATTCCCCATCATAACCAGTGCTTCCTGCTAAATTATATCTGCTTGCCCCTAGATGGACAGGAGTGGATCAGGTGCATGGGTTGTGAAGGGAGGACAAATTACAACCACTTCTCAAGAGTCCATATCCAGATCCAAGAAACAGTTCTTACAGCTGAGCTCTGTGCCCAGTGAACACACAACTACGCATTTTTAAGCAAAAGACACAATGAGGGGACTTCATTATGAGCCTAGACACAAATCTCCCTGCAGGGGTGCATAGGACCAGCAGGGGGCACTCGGGGCAGCATGGGGACTTAGGATAATTGTCGGGGATCAGGATGAGCAGGATCAAGGCTCAGCATTGGGACAGGTGCAAGAGGGCAGAAAAGGGGCTGTAGATGTGGGTTGTTTTCGTCATCATATTTCACCAACAGACACCCTCCACTACATCTCTTCTAATGTATCTGAGTGTTTATATGATTATAAAATGGCATTTATGTAAATACTAATATATACCCATATGAAGGTGCATTGAGTGGTCCTCGCCATCTCATGTGGCCCTTGTCCATCAAGCAATAAGTCCCTGTATTCACTTGAGTACCTCAAACATTACAGTCCAAACATGTAAGGTTTCCCTTTTTCTTGGTCTCTCCTCCTCCCTTCTCTCTCTGTCACACAAACACACATGAACTGACACACACACAGAGCTTCCCAACTTTAATTATCTGATGTATTGAAGCAAACTGATTAATATGCAGCTTTTCTGCTTTGCCTCCCATTTATGTTGTGGTGAAAATAAGAACCATGTGTTTCTCAGCTGGGCACTTCTCTAAGTTAAGTAGCAGCTTTGTTTATTACAACCAGAGAGCAAAAGTAATCCAGGTGTTAGTCAGCAGCTTAAGAGGTCAACAAGTTGTGGAAAATTCATTTACTGGAATAATACCCACTGTTACAATCAAGTACTGTTGGATACACTCAACACCATGCTTAAAATAACAAGTACCTGAATAAGTAAAATAAGTCAAACAAATAAAAGTGCATACATACAATTCCACTTCTATAATTTCTCTGAAGTAAAAATGAACTTAAATAAAAAGGTCTGTAGTTGACTGTGGATGTGGTACAAGAAGAGAAGGTGTGGAAAAGAGAAACTACAGAAGAGCAAAAGGAAATTTCTAGGGTAACTGATTTTTTTCTGTGTTAGTAAAAGTGAGGATTATGTCAATATTTGTAAAATTGTCCACTTTACGTAAAGATTATTATTTGCTAATTTCACCTCATTAAAACATTGCAAATTTTAAAATGTATAATTTGTTAGAAAAGGTGCTAGAGAGAGATTAATAAAATACGTAAAAGTCAGAGACTCCTGAATACACACATGAATGAACCCTGGGTCTCGCTCTACTTTTAGGGAGACACTAGAATACAAAAACATAATGACAGGATTACAGTTCATGAAAGGAGCTTCTCAAACCCCAGGAGGCATGTCCAACTGCGTCCTGGAGTTAACTCAGGGAGCAGGCATGTCCTTTGGAAGGAGCCATGACACCAAGCTCCCAGCATCCATTGTAGCTGACACCATGCAAATGCCAAGAGATCTCAACTAAAATTTTCTGTGGATGTTGAGTCTGATTATGCCACACACTCACACCAAGTGAGTATGGTAAGGATAGTTACCTGCATCCTTAAGGTGTCTGCTGAGAGCAGGGCAGGTCTCTCATTAAGGTCCAAAGTGGCTTGATAAAGCAGGGAAGGAGACTGGCTCAGGGTTGTTATCATGATTTTGTGGTGTGGGCATCCTACTGGCAGGAAGGGTTTTGTGGGGTTTCAAGGTCAATTTGGCATCAAATAAGGGAGCTCCTGTGATTTCTAACTAGATTTATCCTGTGTGCTTTAAAAGAAAGAGAGATGATGGAGGAAAGAGCCTTATGCTATTAGCAATCAGGCATAAAAATAGAACCTGATGACTTACTCTAAATAGCAAGTATAAAAATAATGAGGAAGAAAAGAGACAAGATTCCAATATGGGTGGACAACAACCAGGTCTGCAGAAAATGAGAAGACTGTTTATAAGCAAAGAATAATGAGTAGAAGGAGGATATGAGAAGGATTCTGGTCCAATATCTTGTGTGGAAGCTTTTCATGATTCGAGATCATCAGCTTATTCTGAAGGTCTTAGGTCACCTGTCTTCTTCAAAATATCAGAAGTTCCAGAGGATATGTGAGGATGCACAGTTTAACTTCCTCTATTTGAGTAGCTTTACAATTGTGTAAAATTCTTAATTCTTTTTTTTAAGATGAGTTCTCACTCTGTAGCACAGGTTGGACTGCAGTGGTGTGATCATAGCTCTCTGATTTTAAATTCCTGGCTCATAGAATCCTTCCACCTCAGCCTCCTGAGTAGCAAGTAGCTAGGTCTAGAGGGCCCTTCATCTACACCTGGCTGATTCCTTTTTATTCATTTTTTCATAGAAACAGGGTCTCTCTATGTTGCCCAACCTGCTTTTGAACACCTGGTCTCATGCAATTCTCCAGGCTTGGCCCATCAAAGTGTTGGGATTACAAGTGTAAGCCACCTCATCCAGTAGTCTGCACTGATTTTTTTGTTATAAAGTATGAATCCAATAATTGATTCCCTGGATTTTTTCTGCAGTGCTGTGGTTAAAAGTACCTTATAAGATTCCTTCCAATGTGGCTGAAGAGCAGTGTTTTTCCCTAATGTTTCTTCCAATAGAAATGATTTTCTGGTTGAAGTTCACAAGAAACTGTTAAAAAGATACAGTAAAAAGGCAGCCGTAAACTGTTGGTGATTCGAGTGTATATAATACAGGAATCACTTTCTATATTTTGTAACACATGCATGCAGCAGGAAAAAGGTGAGTGATGAGAGTCAAATCTCTGAATGTATGGGCCTCCCAGATACAATTCATAGGCTAATAACTGACGTGCCCCAAGGAGTGAAATGTGATGCCACACATGCTAGCAGGAGAACACGTGGCCAAGGAAGTTTTTTATAATTTCTATTTAAAAATGTCTATTTCCATTTTTTATTTTTTTTATATTTTCCTACTTTATTCAAAGGAAAGATCTAAAATTGACACCCTAACATCACAATTAAAAGAACTAAAAAAGCAAGTGCAAACACATTCAAAAGCTAGCAGAAGGCAAGAAATAACTAAGATCAGAGCAGAACTGAAGGAGATAGAGACACAAAAAATCCTTCAAAAAATCAATGAGTCCAGGAGCTGTTTTTTTGAAAAGATCAACAAACTTGATAGACCACTAGCAAGACTAACGAAGAAAAGAGAGAAGAATCAAATAGACATAATAAAAAATGATAAAGGGGATATCACCACCGATCCCACAGAAATACAAACTACCATCAGAGAATACTATAAACACCTCTATGCAAATAAACTACAAAATCTAGAAGAAATGGATAAATTCCTGGACACATACACCCTCCCAAGACTAAACCAGGAAGAATTTGAATCTCTGCATAGACCAATAATAGGCTTTGAAATTGAGGCAATAATTAGTAACTAACCAACCAAAAAAAGTCCAGGACAAGACGGATTCACAGCCGAATTCTACCAGAGGTACAAGGAGGAGCTGGTACCATTCCTTCTGAAACTATTCCAATCAATAGAAAAAGAGGGAATCCTCCCTAACTCATTTTATGAGGCCAGCATCATCGTGATACCGAAGCCTGGCAGAGACAAAACAAAAAAAGAGAATTTTAGACCAATATCCCTGATGAACATCAATGCAAAAATCCTCAATAAAATACTGGCAAACTGAATCCAGCAGCACATCAAAAAGCTTATCCACCATGATCAAGTGGGCTTCATCCCTGGAATGCAAGGCTGGTTCAACATACGCAAACCAATAAACGTAATCCAGCATATAAACAGAACCAACGACAAAAACCACATGATTATCTCAATAGATGTAGAAAAGGCCTTTGACAAAATTCAACAACACTTCATGCTAAAAACTCTCAATAAATTAGGTATTGATGGGATGTACCTAAAGATAATAAGAGCTATTTATGACAAACCCACAGCCAATATAATACTGAATGGGCAAAAACTGGAAGAATTCTCTTTGAAAACTGGCACAAGACAGGGATGCCCTCTCTCACCACTCCTATTCAACATAGCATTGGAAGTTCTGTCCAGGGCAATCAGGCAAGAGAAAGAAATAAAGGGTATTCAATTAGGAAAAGAGGAAGTCAAATTGTCCCTGTTTGCAGATGACATGATTGTATATATAGAAAACCCCATAGTCTCAGCCCAAAATCTCCTTAAGCTGATAAGCAACTTCAGCTGAATAACTTTGTATGACTAATAATATAAAATGTTCCACATATTTTTAGCCATGTTTTACTTATTTGTGAAATGTGTGTTTATTAATTTTTCATTTTAAGATGTCACTTTTATCTTTCTTGTTTCTGGGATTTTATTCTAGTAGATATAGCTGAAGGTATTTTAATAATTATTGTAATAATCTCACATTTTATATTTGAATTTTCATATCCATTTCATTAAGATTTTGATTTTGATATAAAATATTTTCTTACAATTGTCTTTTCTATTTTTATGAGATAAAATTAATGTATACAAAATTGCATACATCTTCAATGTACAGTTTCAGGTGTCTGACATATGTGCACACATTTGTCTCCAGCACCTAAGTTAGGATGAGGAGCAGGTCAATCTCCACAACAAGTGTCCTCTTTGGTGCTTCCAGTCAGCTCTCACATAAGGATTTTTATTTAATTTCAAGTTTTCATTCAGATACAGAGGGCATATGTGTGGACTTGTCACATGGGATTATTGAGTGATGCTGAGGTTTGGAATCCAGATTCCATCACCCCCTCCCTCCACTCTCCAGCAGTCCACAGTGTCTATCATTCGCACATTTATGTCCATGAATGCTCAATGCTGAGGTCCCACTTAGATTATGTGGTATTCGGTTTTCTACTCCTGCATTGATTTATTTAGGTTTCAGGCCCCCAGCTCCAATCATTTTGCTGGAAAGGACATGACTATATTATTTTTCATGGCTGTGTAGTGTTATATATTGTAGATGTAACACATTTTGTATATTCAGTCTACCATTGGTGTGTATCTGGGTTGTCTTTGCCACTATGGATAGCACAGCAATGAACATAGATGTGCATGTGTCTTTTTGGTAGAATTGTTTGCTTACTTTTTAGTGTACACCCAGTAGTGGGATTGCTGGGTAAAATGATATCTCTGTTTTAAGTTCTTTGAGAAATCTCCAGTCTGGTTCTCAAAGTGGTAAGACTAATTCATATTCCAATCAACAGTGTATAAGTGTTCTGTTTTCTCCACAGCCCCACCAGCATCCATCGTTTTTCGACTTTTTAGTGATAGCCATTCTGAGTGGTGTGTGGCTTCTCACCTACAGTCATCTCATCTTTGATAAGACTGACAAAAACAAGCAATGAGGAAGGGACTCCCTGTTCAATAAATGGTGCTGGGGCAACTGGCTATCCGTATGATGAAGATTGAAGCTGGACGTCTACTTTCAACACACATAAAATTAACTCAAAATTGACCACAATTTTAAATGTAAGACCACAAACCATAAAAATCTTTTAAGACAACCTAGGAAATACTCTTCTTGACACCAGCTTTGACAAAGAATTTTTGGCTAAGTATCCAAAAGCAATTTCAAGAAAAACAAAAATAGGCAAGTGGGACCTAATTAATTAAGGAGTTACTGCACAGCAAAACAAACACACAAGCAACCAAAACTTCAGCAGATAAGCAGACAACCTGCAAAATGTGAGAAGATATTCACAAACATTGCATCCAACATAGCACTAATATCCAGAATCTATAGGGAACTTAAACAAATCAGAGGCAAAACACAAATAACCCCTTTAAAAATGGGCTGATATGGTTTGGCTGTGTCCCCACCAAAATCTCAATTTGCATTTTATATCCCAGAATTCCTACATGTTGTAGGAAGGATCTAGGGGGAAGTAATTGAATCATGGGGGCCAGTCTTTCTCATGCAATTCTCGTGATAGTGAATAACTCTCATGAGATCTGATGGGTTTATCAGATGTTCCCGCTTTTGCTTCTTCCTCATTTCCTCTTGCCACCACCATGTAAAGAGTGCCTTTCACCTCCCACCATGATTCTGAGGTCCTCCCAGCCATGCAGAACTGTAATTCCAATGAAACCTGTTTTTGTTCCCAGATTTCAGTATGTCCTTACTAGCAGTTTGAAAACAGACTGATACCTGGGAAAATAACACAAACAGACACTTCTCAAAAGAATATATACAAGTGGCCAGCAAATGTATTTTAAAAATATTTAGCATCACTAATCATCAGAGGAATGCAAATAGAAAAATGTTCTGGTTTCTGTCACTATAGGTCCATTTTTTCCTGTTTTGAGCATCACATAAATGGAATCAATATTATAGTTCATTTTTTGTAAGGGGCTACTTTTGGTATTTGTGAGGTTCATTCATGTATTTGCATCTATCTAGGTTTTGTCATCATATATATACATATGTATGTACTCATGCACATCTAGCTATTTCATATCTCAATCAGTCCATTACGTTAATAAACGACAGTTTATTAAGTAAGGAAATCAGTTCATTAAGTGAATAAGTGTCAGTACATATATCTATTTTCCTGTTGATGGAATTTAAATTTGTTTCCAAAATAAATATTGTAAACAAACCTGTTATAAATATCGTCGTACAAGTTCTTTTGCTTATATTCTCCCATTTTTATTGATAAAATATGTAGAAATATAAGTATGCATTATAACTTTTCAGCTTTAAGGAGCTATAACTGACAAACAAAATTGTATGTATTTAAGGTACACCACTTAAGGTATTGATATACTTGGGCACATGTTCATCGTGATCAAGGTAATTGGCATGCCTATCATCTCAGAGAATATCATTTTATGCCTTTAATTTATTGTGAGTCTGTGATAAAAACACCTAAGATCTACTCTTCTGGAAACAGATAAGTTTATAATATAATATTCATTAGTATAGTTGCATTGCTGTATGTTTCATCTCCAGAACTCCTTCAACCTGTGTATCAGTCCATTCTCAAACTACTTTAGAGAACTACCTGAGACTGGGAAATACATGAAGAGAAGTGAATTAGATGACTCACAGTTCTGCAGGCTTAGCAGGAAGCCTTATAGGGAGGCATCAGGAAAATTACGATAATTGTGGAAAGTGAAGGGGAAGCAAGGACCGTCTTCACATGCTGGCAGGGGAAAGAGAGAAAGAGCAAGGGGAGATGCGCCGCACTTTTAAACCTCGAGATCTTGTGAGAACTCTAACACAAGAAGAGCAAAGGGGAAAGCCGCCTCCATGATTCAGTCACTCTTCATCAGACCCCTCCTACAACACTTGAGGATGAAAATTTGACATGAGATTTGGATGGAAACGCTGAGCCAAACAGTATCATTATACCCGGCCACTCACAAATCTCATATCCCTCTCACATTGCAAAATATAATTATCCCTTCTCAACAGTCTCCCAGTTTTCACTCAATTCAGCATTAACACAAACTTCTACAGTCCAAAGTCTCCTCTGAGACAAGGTAAGTTTCTTGGATGTATAACCCTGTAAAATAAAAAACAAGTTAGTTACTTCCAAGATACTATGAGATGAATGCACTGGGTAAATGTTCCCATTCTAAATGGGAGAAATTGGCCACTACAAATGGGCTACAGGCCCCATGCAAGTCCAAACACTAGCAGGGCAGTCATTAAATCTTAAAGCTCCAAAAAAATTTCCTTTTACTCCATGTCTCACATCCAGGACACGCTGATGTAAGAGGTGATCTCCCAAGGCCTTGGGAAGCTCCACCCTTGTGGCTCTGCATGTTAGACTCCCCATAGCTGCTTTCATAGGCTGGCATTGAGTTGCTATGTCTTTTCCAGGCACACCATACAAGCTGCTGGTGGTTCTACCATTCTGGGGTCTGAAGGACAGTGGTCCTCTTCTCACAGATCCACTAGACAGTGCCCAAGTGGGGACTCTGTGTGGGGGATCCAACACCATATTTTCCTTCTACACTGGCCTAGTAGAGGTACTCCATTAGAGCTCAGCCCCTGCATGAGACTTCTGCCTGGATATCCAGGCATTATCATACATCATCTGAAATCTAGGAGGAGGTTCCCAAACCTCAACTCTTGCCTCATGTGCACCAGCAGGCTCAACACCACGTGGAAGCAACCAAAGCTTAGGGCTTGCACCCTCTGGAGCAATGGCCTGAGCTGGACCTTGACCCCTTTTAGCCATGGCTGGCACAGGACAGACAGGGATGCAAGGCGCCATGTCCCAAGGCTGCACAGAGCAGTGGCATCCTGGGCTTGGACAATGAAACCATTTTTCTCCCCTGGGCTTCTGGACCTGTGATGGGAGGGGCTGCCATAAAGATCTCTGAAATGCCCTGGGGACATTTTTTCCCACTGGGGTCCTTGTTACTTATGCAAATTTCTGCAGCCAGCTTGAATTTCTCCCCAGAAAATGAGTTTTTCTTGTCTACCACTTGGACAGGCTGCAATATTTTATCAAACTTTTATGCTCTGCTTCCCTTTTAAACATAAGTTCCAATTTCAGACCATCTCTTTATGAATGCACATGGCTTCTGTTTTCAGAAACAGCCAGGTCAACTCTTCAATGCTTTGGTGCTTAGAAATTTCTTCTGCCAGATATACCTTAAATTATGTCTCTGTAGCTCAACATTTCACCGATCTCTAGGGCAGGGGGAAAATGCCACTAGTCTTTTTGCTAAATCATAGCAAGTGTGACCGTCACTCCAGTTCACAATAAGTTCTTCACCTTCATCTGACACCACCTCAGACTGGACTTCATTGCCCATATCACTATCAGCATTTTGATAAAACCCATTCAACGAGCCTCTAGGCAGTTTCAAACTTTTCCACATCTTCCTGTCTTCTTGTGAGCACTCCAAGCTTTTCCAACCTCTGCCCATTACCCAGTTACAAAGTCACTTCCACATTTTCAAGTATCTTAATAGCAGTATCCCACTCCTGGTGCAAGTTTTCTGTATTAGTCCATTTTCACATTGCTATAAAGACTACCTCAGACTGGGTAATTTATGAAGAGAAGAGTTTTAGTTAACTCTCAGTTCTGCAGGCTAAATGGGAAGCATTATTGGGAGGCATCAGGACGAACAATGATGGTGGAAGGTGAACGGGAAGCAAGAACCTTCTTCACATGGTGGCAGGAGAGAGAGAGCAAGTGAGCGGGGAGGTGCCACACTTTTAAACCATCGGATCTCTTGAGAACTTTATCACGAGAACAGCGAAGAGGAAGACAATCCCATGACCTGATCACTTTATCTTAGGCCCTTCCTTCAACATGCAGGGTCTACAATCTGGCATGAGATTTGAGTCGGAACACGGAGCCAAACCACATTAACCTTCATAACGGGAAGTTTTTACCCTTTGACCAACAATACCCAATTTTTTCCTCCTCCCAGCCCCTGCGACCTACTATTCTACTCTGCTTCCAAGAGCTTGAATATAAAATATTCAATATATAATTCTATATATAAATGAGATCATGCAGCTTTTGTCTTTCTGTGTCTGGCTTATTCCACTTAGCATAATGTTCTCTATTTGTTGCAAATATAAGAATTTCTTTGTTTTTAAAGGCTGAATAATACTCAGTTTTATGTAGGTATAAGCCACATTTTATCTGTTCATTCGTAGATGGACATTGACTCGTTTTCCTTATCTAGACTATTATGAATAATCTCACAATGGACATAGATTTGTCACACTCAGTTTATTTTCTCTAGATGTATGCTCAGAAGTGGGGATGCTCTATGTCCAGTTCACTGAGTAATCTTCATGTTGTTTTTCATACTGGCTGTAATAATTCACATTTTGTTCCAAACCATACATGGATACCTTTGTACCACATATTCAGGTCTTTGTGTAACTCTTGAATCCATTTTTAGCTGATTTCTGTGTATTGTGTGAGGTGAGTTCTATCTATTTCTTCTGCATATGGATACCCGTTTTTCACACCACTCGTTGAAGAGACTGTCCTTTCTCTACTGTGTGTCTTGGGAACTTGACAAAGATCAGTTTATTGGGAAGAAATGGGTTGGTTGCCAGGTTGTGTTTAATGTTTCATTGGATTATATGTCTGTTTAAATGCCAGCATTATAACATTTTGATTTATATAGATTCGATTTTGAAATTATAGATTATGATATATTCAGCTTTGTTATTTATGCCCAAAATTAATTTGGCTATTTGAAGTCTTTTATATTTTTATATAAATTGGAGCTTTTTAAAAACATTTTGTAAAATCATGCCATGGAGATTATTTATTTATTTTATTGGCATATAGTAGATATACCTATTTTATGGGAACATGTAATATATTGATACATTTATAAATGTGTAAAGATTAGTGTAAGATTTACATATCTGTCACATTAAAAATGTACCCTTTCCTTGTGCTGAGCACATTTGAATGACTCCTTTCACTATTTTAATGTTGTGCATTAGATTATTATTAACTATAGTGACCTTACTGATCTATCAAACATTTAGGTCTTATTTCTCCTCTATAAATGTATATTTGTATACAGTAATCAACCTCTCCTTATCTCCTTCTCTCTTCTATCCATCCAGGATTCTGGTAAGCAACAGTCTATTCTATCTTCAGGCGATCCAGTATTTTAGTTTTGACGTAAAATAAGAAGATGCAATATTTGTCTCTCTTTTCTTGGCTTATTTATCTTAACATAATGACTTCCAGTTCCATTCATATTATAGCAAATGACAGAATATTGGGTTTCATGGCTGAATAATATCCTATCGCATACATAGATTACATTTACTCTATCCATTTGTCCACCTACAGACACTGAGGTGCTTTCACATCTGGGCTATTGTGAAAGAGCTGCAAAGAACATGGGAGTGCACATGCCTTCATGAGGTGGTGGTGGTTTTATCTTCTTTAGAACATACCCAGAAGAGGATTTGCCAAGTCATAAGGTATTTCTGTTTTTAATTTATTTTGGAATCTTCATACTACTTTGCACATAGTGGAAACACAAATGGAGTAAACATTAAAAAAGCAGTTTCGGTTTTGATGTATAATCCAGAAACAAATAATGTTTGACCATGATTCTCATTGGGAGTCTCTAATGGATCTGGTGGAAATGCAGGAAGTTTTCTAACCTTGTTCAGGAAATTATGAGTTTGCACCTTTTTCTTTCTGAGGCTTGAAATTGGCATGATTCATGATGCATACTGTTGGGAGGTTTCAGAATGATATGGCAAAAAACATAATGAAAAGGCAAGACAGAGAGAGAGGGAGAGAGAGAGAGTCAGGCCTAGAAAGAGAAAAAATAATTTTCTCAACAGGAGAAAGTGCTGGATATCTGTATCAGTGTTGGGTTTTGCTCATAGACACATCTGTACCGAGTGAGGAACCATGAAGTCAAGGGAGGGGTCTAGAACTTGTTCAAGTGAAGCATCAGCATATTTCTCACAGGCACCAATTTCCACCACATCACAAGGGTGATGCATTTTTGAATGCCATTAAATATGAGTTCACAGTCAGTAATCATGTTTCCATGAAATTCAGTTAAAATACCAAGGGTGTGTCCAGATCACTCATGCACAAATACACAAAATTTGCTTTTTCACGTGTGGCTATCTAGAGACAAAGTGCACTGAGGAGACTTCAGCAGGTTACAGAGATTTGCTTAAGTTTGGAAAACCATAGGAGAGTGCCTTTAAGTGAAGGTTGGTATATAAATTAAATAGGTCAAAATCCCCTCTATGGAGTAGCGTTCCAATTGTGTGGAATTTTTAACTCCTTATTCACGTTATAAAACATAATCCCTGGGACTGACTCCGTGGAGTTTTACTGCTGTGTTGACAAAAATATCTAACAAGCTTCCTTCCAGTGTTTCAAGGGAAGTTTTTCTCTAAATTTTTTCCAATGTATAAATTTTCCTGGCTAAAGATAGTAAAAGGCTGTTAAAAACACAACTGGAATGGCAGCTTTAATCTTTTGCTGATAGGAGTGAGCATAATACATGAATCTCTTTGAGTGCTCTGCTTCATCCAAATACAATAGGTCAAAGGCTAGTACTGGAGGTAAAATTTCCAAGTGTGTATTTTTCCAGCTACCAAGTCATAGGAAAATAACAGATACATCCTTGAGACAGAGGACCATAATGCCACAATGCTGGTAGGAGTACATGTAGACAAGGGAGTTCAAGGGTTTCTTAAAACTGATAATTTTAATGTAAGAATACCATTCTTTTAAACTTATGTGGAAGACTGTGGGAGGTTATAACAATGCCATATTTTAGTATCAGCCCTGCCTCACAGAATCATTTTATAATAGTTCCTTTCATGAGTGTGCTTCACTCGGTTCATGCAAAAACCTGGCAGGTCACAGGCTAAAAACAGAAAATATAGAAGCTTTTAATCAACTGAAAGAGCTGTAGCCTTTGTCAAGGAATTATTGGAAGACACAGAGAACACAGATAGATGATAAATAAAATACATTCATATCCCATTAAGTGTGGAAATTGAGGAAAGTTTACATAAATGTGTCCAAGTGGCAAACTGTTTATTTAATCTGGTAACCAATTTTTCTATAAATTGTAGAGTTGAAATGCAACAAATACATATTTGAAATGATCTGGCACAAATTAAGCAAATATGGTAACTATGTATTTTCACTCATTTTTATTTAAGATCAGCGGCTCAGCATTGATATTTTTGATAATATCTGTGACTCAGCAGCTTTACCTTTTGAGGATATGATCTGGTATGGCAGTTTTCTTAGCTTCAATGTTACCTCTTTTTGCATTGACTTCTACCTTTATATCTGCCAGGAATCTGGGGAAAAGGAGTGCCTGTAAACGTTCCCTAACTTGCCCATTTTGGTGGGTTTTCCAGAATGTGTGAGATGCTTTTTTTTTTTTTTCAAAGCATCCCGAACCCATGCACTGCCCTGAAACATGTTTATTTCCTAGTTTAACAAATTGGCACTTCTAATAAATGCAATCTCTTCTGCCATCTGTGCTGATTTTACATCAGATAGAGGACTGTGTTCTAAAGGAAATCTTATATTAGTAAGAGCAATTTTGTCATTAACCTAGAGTTTTATTATTGAGGTGTGATTCATAACATATAATATTAGGTAGAGGTTCTCAGTGGCAGTGTCTAAATCTCTTGGGTGTACAGTGTCTTCCCCGTTAACATGAAGCATTTACGAGCACAGTCATAGTTTCCAGCCATGCTTCTCCCTGTCTCACAATCACCACAAACTAACTATGACCACAACTGGAACTTGGTGAGTTATTCTTTAATAAGTTTTAAGTTTGTCTTTAATCTTTAAGCCTCTAGATTATTATGTGAGACATAATCTTTATGCCTCTAGATTATTGTGTAAAGCTATTTCAGAAGAAAGTTAAAAAGAACATTTTAACTGACTAAACAACACAGGAAATCATTAATAAAATGCAAAGTGTAGATGTGAGAGGCTCCAGGCCTGGATAATGCAAGAGTTCATGTATGCAGGCAGTTTCTTTCCCCAATTATACAATGATACACCCAGCATGTCAGCTTCATGCCCCATTTGACTCCTATTATGCAAGCGCATGGAAATGACATGCTCAAGGGTCACACACAGATATGAAAACAGGTGGGAGCAGGAGAGGAGACGACTCTGCACTTCTCCTCTGAAGGACCAGGAAAGCCTGGACAGACCATCTCCCCGGCCTCCATGACTGCGCGACGTGCCCACATGGACACTCATCTCTGACAAGATAAGAGGACTCCATTGATGAGGCTGAACATTTTATGATTTAAATTACTAGAGACTTACATTGAGGTTTCAATAACTAATTTTTATAACCCAAATTTACTTACCCCCATGTTGTTACCTCTTTCTTCAGTGAAAAATTGCTTTTGCTGTAATTGAGTTTTAGGAAGATTCCCAATGTTCACAACTGAGCTTCCAAGAGAGTGTCGAGAACAAAAACTGAATGAGGGCAGAGAAACCCATCTTTACTGTGTTCACCACTAAACTCAAGTGGACTCGGCACTGCCTTTGATCACTGTTGCTTCTCCGCAGAGTTCAGGTTTCTATTTCTCACAATTCTAACACAGACTCTCCCTCTCCTCAAAGGTTTGGCCTCCACTGGCTGTAAGGTCACACTGTTCTGAATTTCTTGTCTGAACCATTTTATGAGGGTGGTGATGAGCCATTTAATGGAAATTTTATATCATCTCAGCATGAATCCTATGGGCGGTCACGAATTATTATTCTGATTATGGTAATTGATTTTTCTCAGCATATTCATTACTAGTGATATTCAAAAACCCTTTTTATTTAAATCTTTGATGCTTCATTTTTTGTTGCTATGACACTTTTTCTTCTACTGAGTCTTCCCACTAGCATTATAACATGACCTAATATCCAGGCTCAGTTGTTATATAACAACCACATATGTCAAAAGCTATGCATTCTTTTCACAGCAGACATAATTTTCTCTTTTCTGGAGATGAACACACACTGCTGAGCTACCCCCACTCACAAGAACATATGGAAAATCATGACATTTTTATTTACTTGACTAATAAATTATGTCATTCTCTCTTCAAATTCTTTATCCCCCAGAATGCCATGACAAACTCTTCTGCATCTGTTCAAACCATAAACTCAGAAACCACATGGTGAGTAAAAGCTCACTTGGTTCTGGATATTGGGTCAAGCTCTTCCCCTCCAATGTCCCACAGCATTGGGACCTCAGCCCACCTGTCCAGGTTCTATCAAGAGAGTTTAGCTCCTTCACAGTGAAGGAAACAATTGAGTTAAGAGGGAACCTGCAGAAGATAGACAATATTGAAAACCATTCATATGACAAGGGATTACTATCAAGCATATAAAATAAACTCAACCCAACTGCAAATAATAATGTGGCTAATAATGGAGGAATAACATTAATACATATTTCTCAAAAGAAGAAAAGAAAAAGGGCAGATAGATGATAGATAGATAAATAGATATATTGGTATGTATACATACATATATCGCTAGAAAACACTAATATTCAGGAAAATGCACATTCACAATGAGATGTCTTTTCATCCTTCATCTTTGCTTGAGAGTCAGAAAATCTAAATAAATAAATAAATAAATAAAACAGGAGCTGGCCAGGATTCATAGAAAGGGAAACTCTTATAGACCATTGGTGGAAATGGAAATTAGTGAAGCCATCATGGGAAAAAATAGAACTACCATATAATTCACAATCCAACTGCTGAGTATGTATCTATTTAAATCATTAAAGTAAAAAACTAATATTGAAGAGATACATGTACACCCATGTTTATTGCAGCACTATCCACCATAGCTAACATATGAAATCAACATATGTGTCCATCAACAGATGAATGGATAAATAAAATGTGTTATATTTACACAATGGAATATTGTTCAGTCTTAAAAATGAAATTCTGCTGTTAGAAGCAACATGGATGGAACTGGACACCATCATGTTGAGTGAAACAAGACAGACACAGAAAAATAAATACAGCATTTTCTCAGTGTTATGTGGAAATTTTAAAAAGTTGATCTTCTAGAAGTAAATAATAGAGTAGTGGTTATGAGACGCCGGGAATGGGAGGAGACTGAGAGACAATAAGAGGTTTGTTAACAAACGTATAATTTACAGGCAGATAGGAGGGATGTGCTCCAGTGATCTACAGTGCAGTAGGGTGACTGCTGTTAACAATATAGTGCACATTTTATGTTTACAAGTAGCCAGAAGACAGAAATTTGTATGTTACCAACAAAAAGAAATGTTAGTGTCTAAGCTGATGAATTTGTTCATTGTTCTGATTTAATCATACCACATGGCACACATGCATAGAAATATCACACTGTACCCCATACAGTTATTATGTGCTAACTTTAAAAAATCCTTTAATTAAAACAGATTTTATTTGCATACATTACAAATGCTTCAACACAGAGCCAGGAATAAATACCATTTTTCTTTGAAATGTGAATTTCCTAACAACGTAGTTACATTCATTTGAACCAAACCGTGTATTTGATCATGGTAAGAATAGACAGGCTTATGCATAGAATGATATATTTTAATTTTAGACTTCTACTTAATACCATAAATTCAAATAATTTTAAAACAACTAAGTAAAAACTATAAAGTTGAGGAAATGTGTACGTGGTGTGTGATGTGTGACTTTTTCCTTGCGCACCACTCTGTCCATGGTGGATGTGTGGTGTGGGTGTCTGTGTCTATTTCTGTTTACTCTGCTTGAGGTTCTCTGTGATTCTAGGATCTGTAGTTCAGTGTCTTTCACAAAACTGGGAACGTTCTTAGCCATTATTTCTTTCAAATACTGTCTCTGTATCTACAATTTCTCCTTTCAGATTTAAAATGTACATATACAATACTTTTTAATATTAATGTTTACTTTCTTCACTCTCTTTTCCTTGCACTTTACTCTGTAAAATTTCTAGTGGTATTTTAAGTGAGTGGTTTCATTTAAAAGGTGAGCCAGCTCTACTGAAGGGTGTGCCCAAAGCTTACTCAATGTTTATACTGCATTGCTTTTGATTTCTTATGCATTTCCATTTGATTTTTTCTTAGTATTTTCAACTCTCAGTTCCCTATCTAGTCCTTCATTATGTATACAGTTTCCTTAATAGATTTTTACATATGAATTATAGTTACTTTATATATCTTGTTTAATTAGATAGTTCTAAGATCCATATCATATAGAAGTCTCATTCTGATCATTTGTTTATTATGACTTTGGTATTTCTCATTAATATATGTAATCTTTGTTGATAGCCAGATATTTTAGGTTGGACAGTTGATATTGGCTTATTACTTCATTTTATGCATTTTCTGCCTGTATTTGACCATACTTTATTTTTGCCAGGCCTTTAATGTGGAAATGTTTGAATCTTCTCAGAGCTACATTTGACGTTTACTTTTGCAGTAGACGTCATAGTTGAAGTCTGTTCTTCTGTGTCCACCAGAGACTTCAGATCCTCCAGTGATACCTTGTTTTTCTTTCCTGCTTGGCTTTGTCTCTTCACCCTGTTCCTTCCTCCAGAGAATCTCTTTCAGCTCCTTCAGGTGGGTTAAGATATTATATTGAACTGACAATTGTGAAATTGGTGGAAGGCAATAGAATAAAGGGAGATTTTCTGACCTTTCATGGGTCTATATTTCTACGAAGGCATTGTGACCCTGAGTCTGGGTGTGACATTGCCGGTGTTTCTGAACTTCTGCCAGATGAGATGTTGGTCTGTGTGTTCTTGCTGTTTTCCCTGCTGTGGAGTCCTCTTGTTTTCCCCAGTTGTTCCCTCCCACAGCTCCAATGTTCTCTTTTGGTGTTATCAGCTTCCAGAGTTGATGACCTGACCTAGAGATTAAGGCTCTGATTAAATAAGAAGGAGGGGAGATACTTCTCAATGGAACTTAGGTGAAGACCTCTTTTTCCATCTCAGTTCCTAAGGGATGGCCCCAGTGCCCCTAAGATCCTGGCTTTGGTGGCTTGCTCCTGCAGAGTAATTTCTTAGTTCTGCAATGGGGATTAAGGAGGTGGGTCTGAATGCATTTCAGAGTGTGGGCTCTTTTTCTCTCCCAGACAGACACATTGGGACAAAAGATTTTTGTGACTGTCCCCATTTTTGGGAAAAAGGTTTCAAGGTATAGGAAAGCTCTTCAGTATGTGGTCCCTGAGAAATTCACGCTACAACACATTTACCACACTTGACTTCACCAATTCAGTATCTATGTATTTTTTTCTCTTATAATAGCCTACATTTTTATATGCCAGACTCTGCCTCAGTTCAACTCGTACCCATGCCTTTTTACTCTCTGCAAGAACTTGTCTCTCCCTAAATTTCGGATTTGATGATTATCTTAAAACCTCCAGTATTTAAAGTATATTTTAAAATTGGCAAAATTCCATCTCCTGTGTTTATCTGTTATGTTGATGCTGTAAAACAGTAAGTAAAATATACTCCTCATCTATGTACATTTTGAAGCTGAGTTGCAGGTTTTTTGGTAAGACCCAGAGTCACAGAGAATTCAAATATTGTTAAGCTGCTTAATAGAAAAACAAATTATGGTAAATGTGTTCACTGGAATACTACCCATGATTTATAATAAATAAATGCCTGACACACAGAACAGCAGCAAAACCACACATGCTCTTATTACAGAAAGTGGCTTCTGAAAACCACACCGGGCATGTACAGCTTTGTCCTGGAGTTGGTTTAGGGGGATGTCAGAGCCAGTGACGAGAAGCACAGGGCCAGATGGCAGCGTTCACTCATCCCAGACATGAGCTCCTGGGTGCATACAGAGCCCCCCCATGTGTGGGTTTACTTCCACTTCTGTAAAAGGAGAAAATACTGACTCCTACAGAGCATAATTTACACATTTTTTAAAAAATGTAATAGGGTGATCAGGGCAAAGTGTTTATCACAGCACAATTTCATAAGACAGCATATTTTCCAAATACCATCATTGTCAGCAAACTTCTGCAGAGCACCGTCTTCTTATATGGGTACAGCCTATTCCTCCAGCATCCCACTAGAGCTTCTTATATAGTAGGAGACATGCAAATAGGGCCCTCCCTCTACTGATGAAAACCAACCCAACCCTGACCCTGCAGGTCTCAGAGAGGAGCCTTAGCCCTGGACTCCAAGGCCTTTCCACTTGGTGATCAGCACTGAGCACAGAGGACTCACCATGGAGTTGGGGCTGAGCTGGGTTTTCCTTGTTGCTATTTTAGAAGG

>CBT15_circR_23615

GACCTGAGCTCCTTCGCCATGCCGCTCCTGGACGGAGACCTGGAGGGTTCCGGAAAGCATTCCTCTCGAAAGGGTGGACAGCCCCTTCGGCCCGGGCAGCCCCTCCAAAGGGTTCTTCTCCAGAGGCCCCCAGCCCCGGCCCTCCAGCCCCATGTCTGCACCTGTGAGGCCCAAGACCAGCCCCGGCTCTCCCAAAACCGTGTTCCCGTTCTCCTACCAGGAGTCCCCGCCACGCTCCCCTCGACGCATGAGCTTCAGTGGGATCTTCCGCTCCTCCTCCAAAGAGTCTTCCCCCAACTCCAACCCTGCTACCTCGCCCGGGGGCATCAGGTTTTTCTCCCGCTCCAGAAAAAG
