# Supplementary figures and images for: RNA sequencing reveals the expression profiles of circRNA and indicates that circDDX17 acts as a tumor suppressor in colorectal cancer
Source: J Exp Clin Cancer Res. 2018 Dec 27;37:325. doi: 10.1186/s13046-018-1006-x (PMC6307166; doi:10.1186/s13046-018-1006-x)

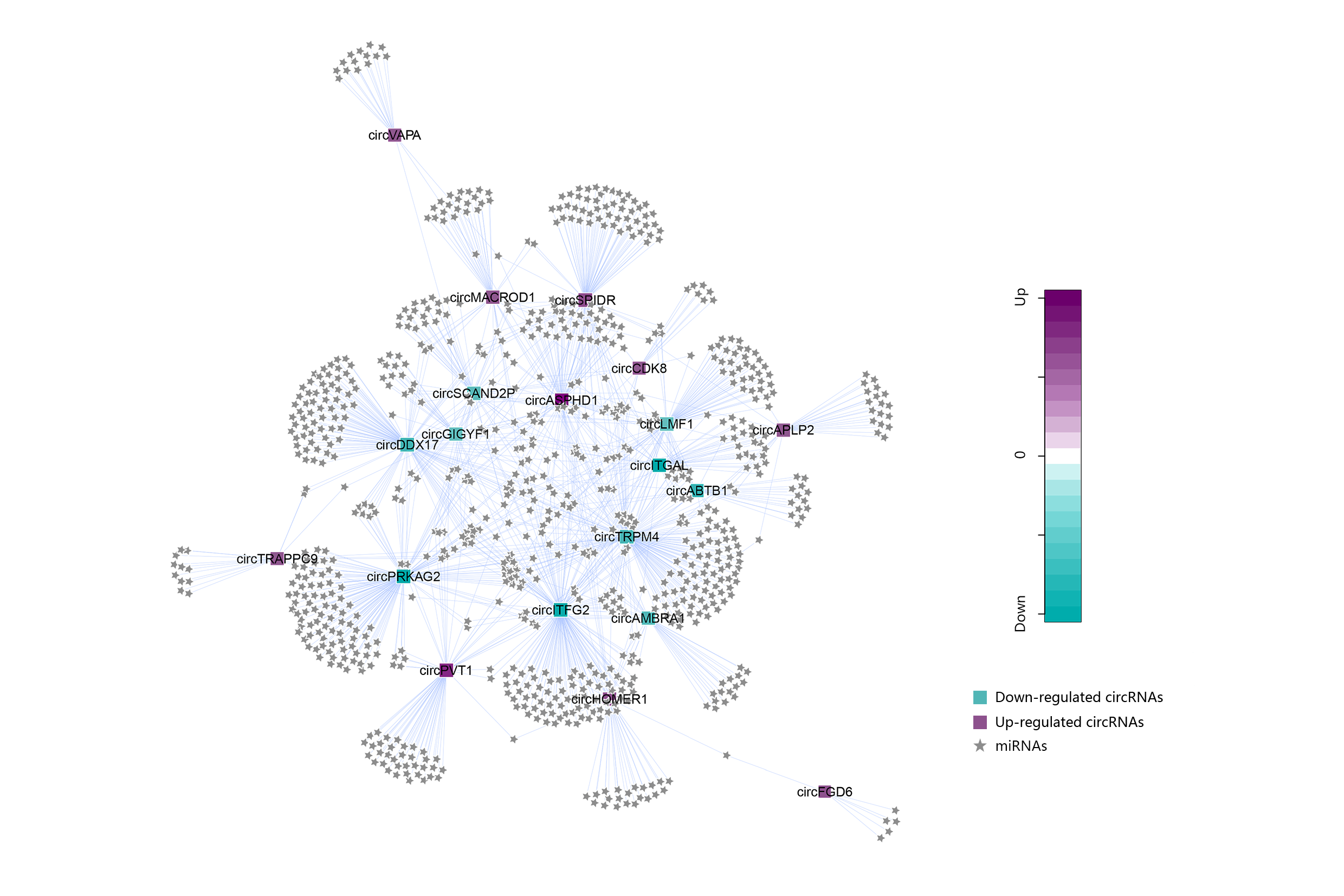

Supplement: Supplementary file 5 — Figure S1. Network of circRNA-miRNA interactions in CRC. The network was based on the top 10 dysregulated circRNAs in CRC and their predicted target miRNAs. The purple square node represented up-regulated circRNAs. The cyan square node represented down-regulated circRNAs. The star node represented miRNAs. The log2FC was represented by a color scale, increased from cyan (relatively lower log2FC) to purple (relatively higher log2FC). (TIF 989 kb) [file 13046_2018_1006_MOESM5_ESM.tif]
